# *Latexin* deficiency limits foam cell formation and ameliorates atherosclerosis by promoting macrophage phenotype differentiation

**DOI:** 10.1038/s41419-024-07141-3

**Published:** 2024-10-18

**Authors:** Guozhang He, Yuanting Ni, Rong Hua, Huaibin Wan, Yanhui Tan, Qiwei Chen, Shaohua Xu, Yuzhong Yang, Lijun Zhang, Wei Shu, Ke-Bin Huang, Yi Mo, Hong Liang, Ming Chen

**Affiliations:** 1 State Key Laboratory for Chemistry and Molecular Engineering of Medicinal Resources, Key Laboratory for Chemistry and Molecular Engineering of Medicinal Resources (Ministry of Education of China), School of Chemistry and Pharmaceutical Sciences, Guangxi Normal University, Guilin, China; 2 Department of Scientific Research, Maternal and Child Health Hospital of Guangxi Zhuang Autonomous Region, Nanning, China; 3 Heyuan Research Center for Cardiovascular Diseases, Department of Cardiology, the Fifth Affiliated Hospital of Jinan University, Heyuan, Guangdong China; 4 Department of Pathology, Affiliated Hospital of Guilin Medical College, Guilin, China; 5 College of Intelligent Medicine and Biotechnology, Guilin Medical University, Guilin, China; 6 Biobank department, The reproductive hospital of Guangxi Zhuang autonomous region, Nanning, China

**Keywords:** Diseases, Lipids

## Abstract

Latexin (LXN) is abundant in macrophages and plays critical roles in inflammation. Much is known about macrophages in atherosclerosis, the role of macrophage LXN in atherosclerosis has remained elusive. Here, the expression of LXN in human and mouse atherosclerotic lesions was examined by immunofluorescence and immunohistochemistry. *LXN* knockout and *LXN/ApoE* double-knockout mice were generated to evaluate the functions of LXN in atherosclerosis. Bone marrow transplantation (BMT) experimentation was carried out to determine whether macrophage LXN regulates atherosclerosis. We found that LXN is enriched in human and murine atherosclerotic lesions, mainly localized to macrophages. *LXN* deletion ameliorated atherosclerosis in *ApoE*^*-/-*^ mice. BMT demonstrate that deletion of *LXN* in bone marrow protects *ApoE*^*-/-*^ mice against atherosclerosis. Mechanistically, we found that LXN targets and inhibits JAK1 in macrophages. *LXN* deficiency stimulates the JAK1/STAT3/ABC transporter pathway, thereby enhancing the anti-inflammatory and anti-oxidant phenotype, cholesterol efflux, subsequently minimizing foam cell formation and atherosclerosis. Gene therapy by treatment of atherosclerotic mice with adeno-associated virus harbouring *LXN*-depleting shRNA attenuated the disease phenotype. In summary, our study provides new clues for the role of LXN in the pathological regulation of atherosclerosis, and determines that LXN is a target for preventing atherosclerosis, which may be a potential new anti-atherosclerosis therapeutic target.

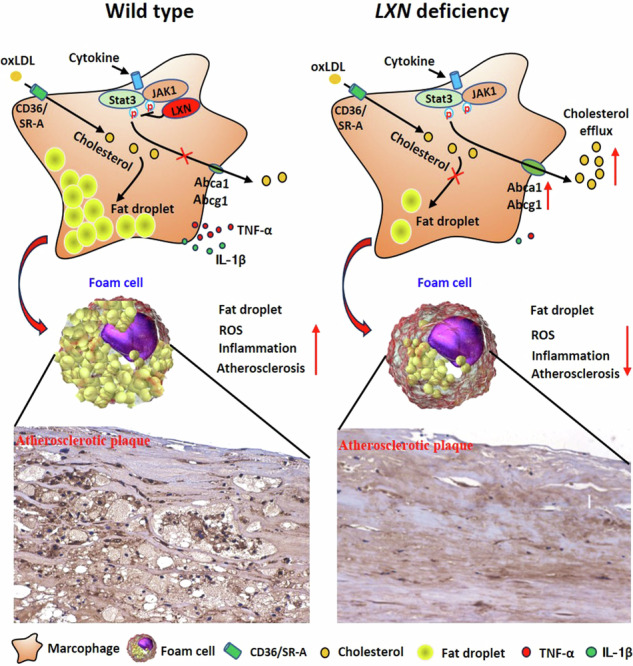

## Introduction

Atherosclerosis is the common pathological basis of cardiovascular and cerebrovascular diseases and the main cause of death worldwide [1], which causing about 17 million deaths every year [2–4]. In essence, atherosclerosis is an arterial vasculitis disease, accompanied by lipid metabolism disorder and inflammatory cell recruitment to the arterial wall [5]. As well known that macrophages play a key role in atherosclerotic plaque formation [6, 7]. For example, macrophages are recruited into the intima and take up modified low-density lipoprotein (LDL) particles with the progression of atherosclerosis; the accumulation of lipids in macrophage results in foam cell formation, which comprise the major cellular component of atherosclerotic lesions [7–10]. As foam cells become overwhelmed with lipids, they undergo cell death, plaque rupture, and subsequent arterial thrombosis and myocardial infarction [11, 12]. Importantly, macrophages have also evolved mechanisms for eliminating cholesterol from the cell, that is, through cholesterol efflux and reverse cholesterol transport (RCT), which significantly prevents atherosclerosis [13, 14]. In this regard, ATP-binding cassette transporters (such as ABCA1 and ABCG1) mediate cholesterol efflux from macrophages, thus limiting the formation of foam cell and atherosclerosis [15–18].

Latexin (LXN) is a carboxypeptidase inhibitor, which was first discovered in the lateral neocortex of rats [19–21], and widely expressed in various tissues and cells, such as the intestine, lymphoid organs, adipose tissue, endothelial cells, hematopoietic stem cells, mast cells and macrophages [22–24]. Previous studies have shown that LXN plays important roles in stem cell. Liang *et al*. believe that *LXN* is a stem cell regulatory gene, and its expression is negatively correlates with the number of haematopoietic stem cells [25]. We also reported that *LXN* is dramatically increased during adipose stem cell differentiation [22]. Recently, increasing evidence that LXN is related to inflammation and immunity has been reported [26–28]. The expression of *LXN* in macrophage is significantly induced by lipopolysaccharide (LPS), while higher levels of *LXN* mRNA in M1 macrophage, but not in M0 and M2 macrophages, have been observed, implicating the potential role of *LXN* in regulating macrophage function [28–30]. We also reported that *LXN* regulates macrophage polarization, contributing to cancer immune escape [26]. Because macrophages play a key role in atherosclerosis, we speculate that *LXN* plays critical roles in atherosclerosis.

In this study, we report a new physiological function of *LXN* in atherosclerosis through mechanisms involving modulation of the macrophage anti-inflammatory phenotype and cholesterol efflux. We show that LXN increases in atherosclerotic lesions, and colocalizes with macrophages in human and murine atherosclerotic plaques. Both genetic ablation of *LXN* and BMT with *LXN*-deficient hematopoietic cells improved atherosclerosis in *ApoE*^*-/-*^ mice. LXN interacts with JAK1 and inhibits JAK1/STAT3 activity, while deleting *LXN* promotes the STAT3/ATP-binding cassette transporter signalling cascade, thereby enhancing the anti-inflammatory phenotype and cholesterol efflux, subsequently minimizing foam cell formation and inflammatory responses. Our findings underscore the function of LXN as a critical regulator of atherosclerosis, and identify macrophage LXN as a potential novel therapeutic target against atherosclerosis.

## Results

### LXN exhibits increased expression and colocalizes with macrophage in atherosclerotic lesions

**Fig. 1:**
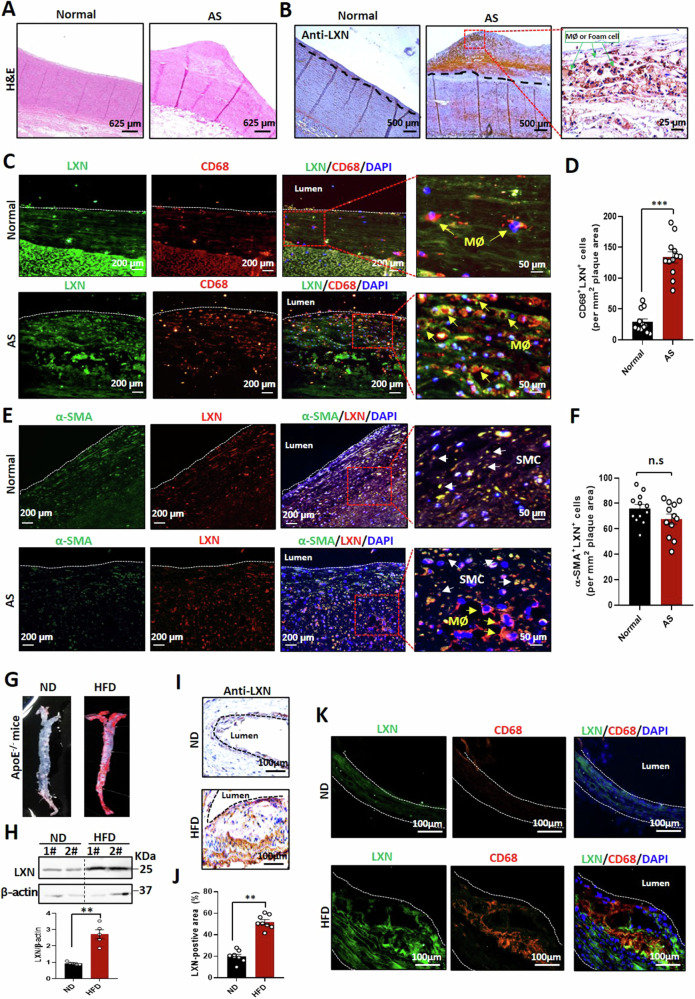
LXN is abundant in human and murine atherosclerotic plaques. **A** H&E staining of normal and atherosclerotic human aorta samples. **B** Immunohistological analysis of LXN expression in normal and atherosclerotic human aorta samples. The red dotted box area is enlarged in the inset. Green arrows, macrophages or foam cells. **C** Immunofluorescence staining of LXN (green) and CD68 (red) in normal and atherosclerotic human aorta samples. The red dotted box area is enlarged in the inset. Yellow arrows, macrophages. **D** Quantification of CD68^+^LXN^+^ cells in human aorta samples (n = 12). **E** Immunofluorescence staining of LXN (red) and α-SMA (green) in normal and atherosclerotic human aorta samples. The red dotted box area is enlarged in the inset. White arrows, SMCs. **F** Quantification of α-SMA^+^LXN^+^ cells in human aorta samples (n = 12). **G** Representative image of *en face* Oil Red O-stained aortas from *ApoE*^*-/-*^ mice fed normally or on a HFD. **H** The expression of LXN in aortas of mice fed normally or on a HFD, as assessed by western blot analysis (n = 5). **I** Representative image of immunohistological analysis of LXN in aortas of mice fed normally or on a HFD. **J** Quantification of the LXN-positive areas in mouse aorta samples (n = 8). **K** Immunofluorescence staining of LXN (green) and CD68 (red) in aortas from mice fed normally or on a HFD. All the data are presented as mean ± SEM. The 2-tailed unpaired Student t-test was used for (**D**, **F**, **J**). Mann-Whitney U test was used for (**H**). **P < 0.01, ***P < 0.001, n.s, no significance.

To begin to understand the function of LXN in atherosclerosis, we examined its expression in atherosclerotic lesions. Paraffin-embedded human aortic autopsy specimens harbouring non-atherosclerotic and atherosclerotic lesions were obtained from Guilin Medical College (Fig. 1A). We performed immunohistochemical staining with an antibody against LXN on the specimens. LXN exhibited enhanced accumulation in atherosclerotic lesions, compared with non-atherosclerotic aorta sections (Fig. 1B). To identify the cell types in the arterial intima expressing LXN, we performed immunofluorescence experiments, staining the arteries for LXN, CD68 (macrophage marker) and α-SMA (SMC marker). Our results demonstrate that LXN was highly expressed in macrophages (as identified by CD68 positivity; Fig. 1C, Supplemental Fig. S1), the number of LXN-expressing macrophages (CD68^+^LXN^+^ cells) in atherosclerotic lesions was remarkably increased compared with that in non-atherosclerotic arteries (AS *versus* Normal: 133.8 ± 31.22 *versus* 28.9 ± 17.9, *P* < 0.001) (Fig. 1D). LXN was also expressed in vascular SMCs (marked by α-SMA; Fig. 1E). However, the number of LXN-expressing SMCs (α-SMA^+^LXN^+^ cells) did not significantly change between normal arteries and those with atherosclerotic lesions (Fig. 1F).

We also determined LXN levels in mouse atherosclerosis. To this end, HFD was used to feed atheroprone *ApoE*^*-/-*^ mice for 12 weeks (Fig. 1G), and aorta was isolated for determination of LXN protein levels. Western blot and immunostaining show that the level of LXN in atherosclerotic aorta was >2.5-fold higher than that in normal controls (Fig. 1H–J). We also detected the expression of LXN in normal blood vessels, and it is worth noting that, LXN was rarely expressed in normal vascular intima, but significantly increased in atherosclerotic plaques, suggesting that the increased LXN in atherosclerotic plaques did not originate from endothelial cells. To clarify the origin of LXN within the plaque, mice normal aortas were whole mounted and stained with CD31 and LXN antibody and were examined by confocal microscope with Z-stacks model, we proved that LXN was mainly distributed in the vascular endothelial layer in normal aorta vessels (Supplemental Fig. S2A, B). Interestingly, we found that LXN was almost exclusively expressed in endothelial cells (labeled by the marker CD31) in normal aorta, however, abundant of LXN invaded into the intimal layer at the plaque site in atherosclerosis mice (Supplemental Fig. S2A) and were significantly correlated with areas of aortic plaques in *ApoE*^-/-^ mice (Supplemental Fig. S2C). More importantly, immunofluorescence analysis revealed that LXN colocalized with macrophages (labeled by the marker CD68) in aortic lesions (Fig. 1K), indicating that the increase of LXN in atherosclerotic plaques is mainly caused by infiltrating macrophages rather than endothelial cells, which further suggested that macrophages LXN could play an important role in the formation of atherosclerosis. Taken together, these data obtained from human and mouse model demonstrate that LXN levels are specifically increased and colocalized with macrophage in atherosclerotic lesions.

### Global *LXN* deficiency protects *ApoE*^*-/-*^ mice against atherosclerosis

**Fig. 2:**
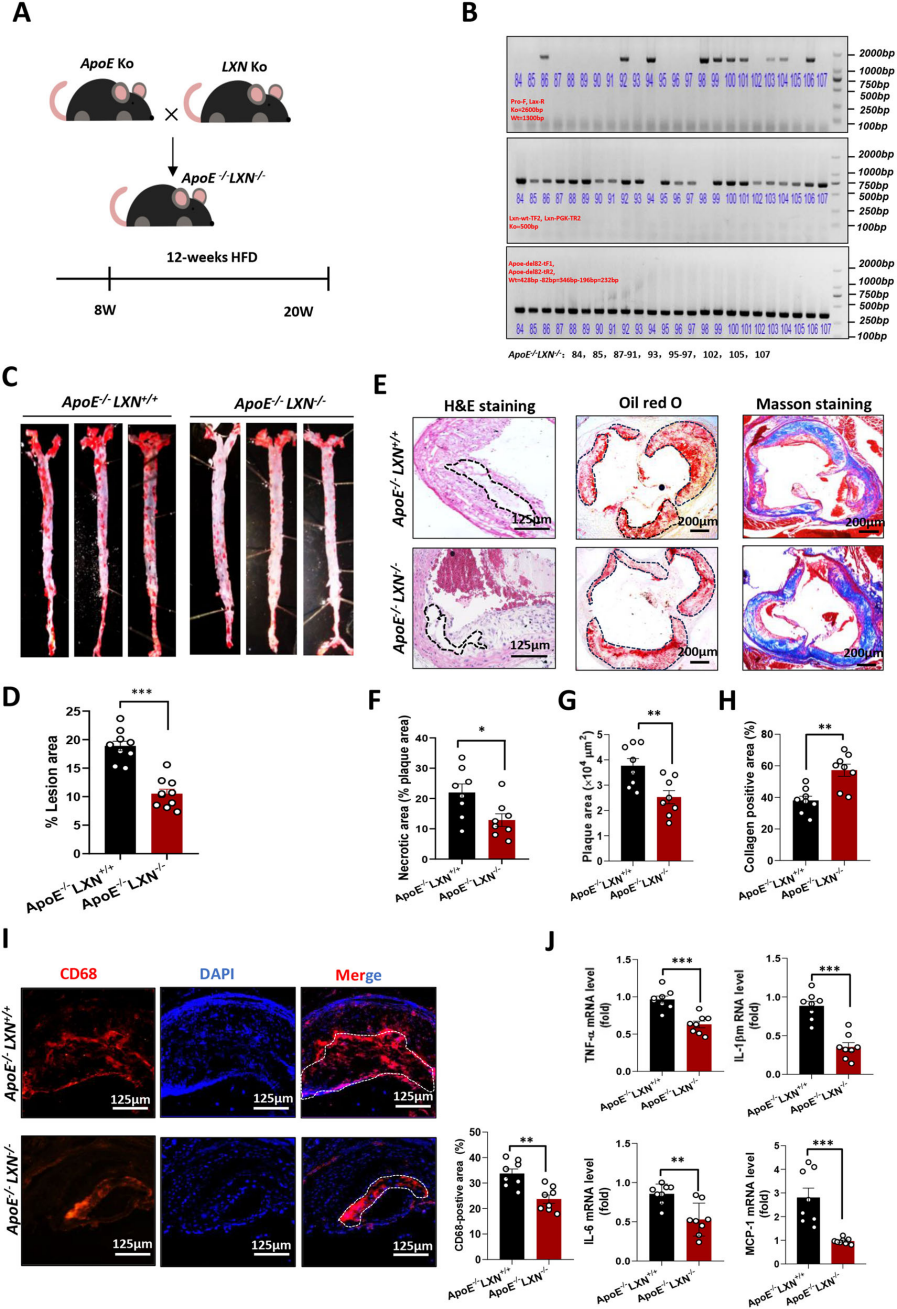
Global *LXN* deficiency suppresses atherosclerosis in *ApoE*^*-/-*^ mice. **A** Schematic diagram of the strategy for generating an atherosclerotic *LXN/ApoE*-double knockout mice. **B** Genotypic identification of hybrid mice. **C** Representative *en face* Oil Red O staining of aorta. **D** Quantification of plaque area in aorta (*n* = 10). **E** Representative images of staining performed on aortic root cross-sections. **F–H** Necrotic area (**F**) (*n* = 8), lipid deposition (**G**) (*n* = 8), collagen content (**H**) (*n* = 8) in aortic root sections. **I** Representative images and quantification of CD68 in atherosclerotic lesions of HFD-fed mice with the indicated genotypes (n = 8). **J** mRNA levels of *TNF-α, IL-1β, IL-6*, and *Mcp-1* in atherosclerotic plaques from HFD-fed *ApoE*^*-/-*^*LXN*^*+/+*^ and *ApoE*^*-/-*^*LXN*^*-/*-^ mice, as determined by qPCR (*n* = 8). All the data are presented as mean ± SEM. The 2-tailed unpaired Student t-test was used. *P < 0.05, **P < 0.01, ***P < 0.001.

Next, we investigated the function of LXN in atherosclerosis. To this end, we constructed a global *LXN*-deficient mice [22], and hybridized it with *ApoE*^*-/-*^ mice to generate *LXN/ApoE* double-knockout mice [24], and then fed HFD for 12 weeks to induce atherosclerosis (Fig. 2A, B). The plasma triglycerides (TG) and total cholesterol (TC) levels in *ApoE*^*-/-*^*LXN*^*+/+*^ and *ApoE*^*-/-*^*LXN*^*-/-*^ were determined. We found that *LXN* knockout had no effect on plasma TG, TC, and LDL-C levels in normal-diet fed *ApoE*^*-/-*^ mice, but dramatically elevated plasma HDL-C levels in *ApoE*^*-/*-^ mice fed with high-fat diet (Supplemental Fig. S3). In addition, we found that plasma TNF-α, IL-6, and IL-1β levels were lower in *LXN* knockout mice, but IL-10 levels were higher in *ApoE*^*-/-*^ mice fed with HFD (Supplemental Fig. S4). Oil Red O staining revealed that the plaque area in *ApoE*^*-/-*^*LXN*^*-/-*^ mice decreased significantly when compared to *ApoE*^*-/-*^*LXN*^*+/+*^ mice (Fig. 2C, D). Additionally, the necrotic core area was reduced in *LXN/ApoE* double-knockout mice compared to *ApoE*^*-/-*^*LXN*^*+/+*^ mice after 12 weeks of HFD (*ApoE*^*-/-*^*LXN*^*-/-*^ *versus ApoE*^*-/-*^*LXN*^*+/+*^: 12.87 ± 4.32 *versus* 21.94 ± 6.38, *P* < 0.05) (Fig. 2E, F). The cross-sectional analysis of the aortic root showed that the absence of *LXN* significantly decreased lesion area (Fig. 2E, G), indicating that deletion of *LXN* attenuates the progression of atherosclerosis. Masson staining shows that the loss of *LXN* also increased collagen content (Fig. 2E, H) and the fiber cap area of atherosclerotic lesions in *ApoE*^*-/-*^ mice by more than 40% (Supplemental Fig. S5). A more detailed assessment of the composition of aortic root plaques indicated that *ApoE*^*-/-*^*LXN*^*-/-*^ mice had less CD68^+^ macrophage infiltration (Fig. 2I). Notably, inflammatory factors, such as *TNF-α*, *IL-1β*, *MCP-1* and *IL-6*, were decreased significantly in plaques in *ApoE*^*-/-*^*LXN*^*-/-*^ mice (Fig. 2J), indicating that *LXN* deficiency attenuates the inflammatory response in plaques. Collectively, our data reveal that global *LXN* deletion protects *ApoE*^*-/-*^ mice against atherosclerosis.

### Bone marrow-specific *LXN* deficiency reduces atherosclerotic lesion formation

**Fig. 3:**
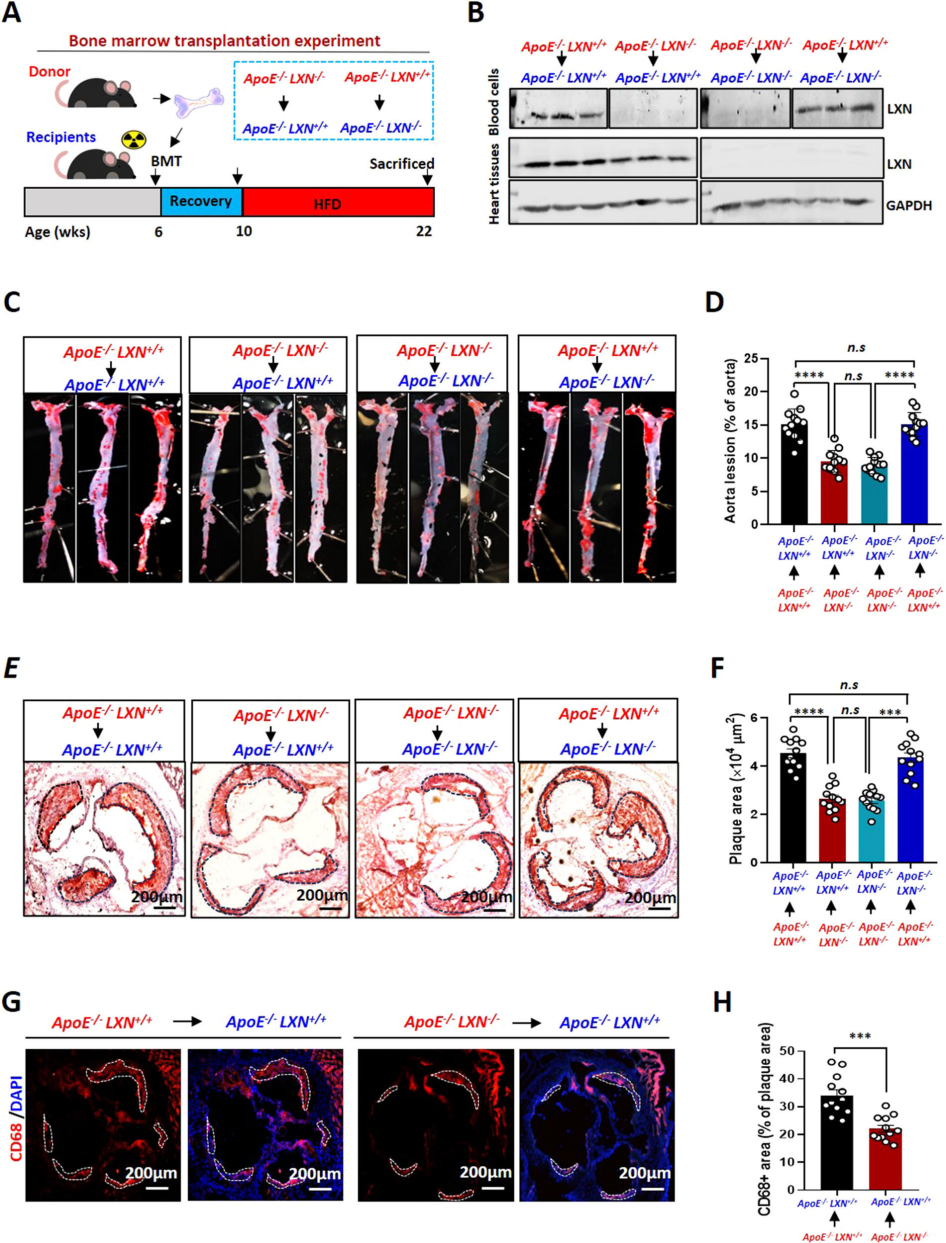
Deletion of *LXN* in hematopoietic cells alleviates atherosclerosis in *ApoE*^*-/-*^ mice. **A** Schematic diagram of the BMT and genotyping strategy. **B** Successful reconstitution of recipient bone marrow with donor bone marrow, as verified by western blot analysis of LXN levels in blood cells. **C,** **D** Representative images of *en face* Oil Red O-stained aortas from the indicated groups (**C**), and quantification of the lesion areas (**D**) (*n* = 12). **E,** **F** Representative images of cross-sections of Oil Red O-stained aortic roots from the indicated groups (**E**), and quantification of the plaque area (F) (*n* = 12). (**G,** **H**) Representative image (**G**) and quantification (**H**) of CD68 in atherosclerotic lesions in control mice (*ApoE*^-/-^*LXN*^+/+^) and *ApoE*^-/-^*LXN*^+/+^mice transplanted with *ApoE*^*-/-*^ *LXN*^*-/-*^ BM (*n* = 12). All the data are presented as mean ± SEM. One-way ANOVA was used for (**D**, **F**). The 2-tailed unpaired Student t-test was used for (**H**). **P < 0.05, ***P < 0.001, ****P < 0.0001, n.s, no significance.

Given our findings that LXN is enriched in plaques and colocalizes with macrophages in atherosclerotic lesions, we further determined whether LXN expression in macrophages regulates atherosclerosis in *ApoE*^*-/-*^ mice. To this end, we constructed a bone marrow chimeric mouse model via transplanting bone marrow (BM) from *ApoE*^*-/-*^*LXN*^*-/-*^ mice to *ApoE*^*-/-*^*LXN*^*+/+*^ mice treated with lethal doses of radiation, or vice versa. Homologous transfers of BM served as controls (*ApoE*^*-/-*^ *LXN*^*+/+*^BM into *ApoE*^*-/-*^ *LXN*^*+/+*^ mice, *ApoE*^*-/-*^ *LXN*^*-/-*^BM into *ApoE*^*-/-*^ *LXN*^*-/-*^ mice). After a 4-week recovery, these bone marrow transplant mice were fed a high-fat diet for 12 weeks (Fig. 3A). Successful reconstitution of recipient bone marrow with donor bone marrow was verified by western blot analysis of blood LXN levels (Fig. 3B). *En face* analysis of Oil Red O-stained sections revealed that the atherosclerotic plaque burden was decreased about 40.4% in the *ApoE*^*-/-*^*LXN*^*+/+*^ mice that received *ApoE*^*-/-*^*LXN*^*-/-*^BM compared with *ApoE*^*-/-*^*LXN*^*+/+*^mice that received *ApoE*^*-/-*^*LXN*^*+/+*^ BM. In contrast, the atherosclerotic plaque burden was increased in *ApoE*^*-/-*^*LXN*^*-/-*^ mice that received *ApoE*^*-/-*^*LXN*^*+/+*^BM compared with the corresponding control group, whereas no significant difference in atherosclerosis was observed in different background mice with identical bone marrow transplantation (Fig. 3C, D). The atherosclerotic burden in the aortic root was in line with these findings (Fig. 3E, F). Notably, *ApoE*^*-/-*^*LXN*^*+/+*^ mice that received *ApoE*^*-/-*^*LXN*^*-/-*^ BM displayed decreased CD68^+^ macrophages in atherosclerotic lesions compared with the corresponding control group (37.225 ± 5.17 *versus* 22.71375 ± 3.83, *P* < 0.001) (Fig. 3G, H). These results suggest that bone marrow-derived cells loss *LXN* attenuates atherosclerosis. As macrophages are the key component of atherosclerotic plaques, and mostly originate from bone marrow, we can conclude that *LXN* deficiency in macrophages is sufficient to reduce atherosclerotic plaque formation.

### Macrophage *LXN* deficiency promotes an anti-inflammatory phenotype and protects macrophages from oxLDL-induced oxidative damage

**Fig. 4:**
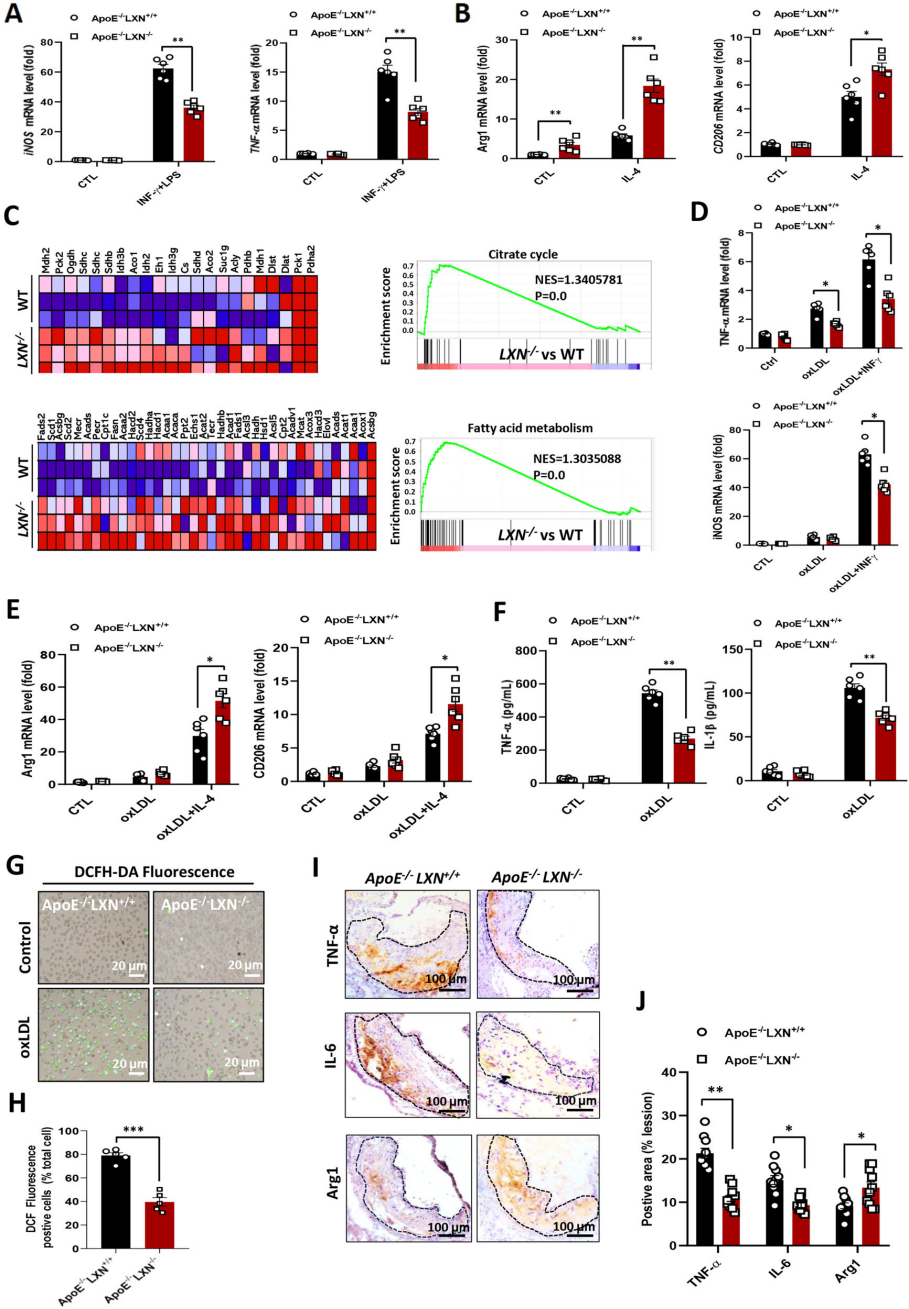
*LXN* deficiency enhances M2 polarization and protects against oxLDL-induced inflammation and oxidative damage. **A** Representative qRT-PCR analysis of M1 macrophage markers in *ApoE*^-/-^*LXN*^+/+^ and *ApoE*^-/-^*LXN*^*-/-*^ BMDMs after induction with IFN-γ + LPS (30 ng/mL + 100 ng/mL) for 48 h (*n* = 6). **B** Representative qRT-PCR analysis of M2 macrophage markers in *ApoE*^-/-^*LXN*^+/+^ and *ApoE*^-/-^*LXN*^*-/-*^ BMDMs after induction with IL-4 (20 ng/mL) for 48 h (*n* = 6). **C** GSEA of RNA-seq data from the BMDMs of LXN KO versus WT mice using the signalling pathways regulating cell metabolism, including pathways in the citrate cycle and fatty acid metabolism presentation gene set annotated in the KEGG database. NES, normalized enrichment score. **D** mRNA levels of *TNF-α* and *iNOS* in *ApoE*^-/-^*LXN*^+/+^ and *ApoE*^-/-^*LXN*^*-/-*^ BMDMs incubated with 20 μg/mL oxLDL or oxLDL + IFN-γ (20 μg/mL + 30 ng/mL) for 48 h, as determined by qRT-PCR (*n* = 6). **E** mRNA levels of *Arg*1 and *CD206* in *ApoE*^-/-^*LXN*^+/+^ and *ApoE*^-/-^*LXN*^*-/-*^ BMDMs incubated with 20 μg/mL oxLD or oxLDL + IL-4 (20 μg/mL + 20 ng/mL) for 48 h, as determined by qRT-PCR (*n* = 6). **F** Levels of the inflammatory markers, TNF-α and IL-1*β*, secreted from *ApoE*^-/-^*LXN*^+/+^ and *ApoE*^-/-^*LXN*^*-/-*^ BMDMs incubated with 20 μg/mL oxLDL for 48 h, as determined by ELISA (*n* = 6). **G,** **H** the production of ROS in the cells, as determined using a DCFH-DA kit (**G**) and quantification of the ROS, by fluorescence intensity (**H**). **I,** **J** Representative immunohistochemistry images of atheroscle lesions from HFD-fed *ApoE*^-/-^*LXN*^+/+^ and *ApoE*^-/-^*LXN*^*-/-*^ mice, stained with anti-TNF-*α*, IL-6 or Arg1 antibodies (**I**), and quantification of TNF-*α*, IL-6 and Arg1 expression levels in in the indicated groups (*n* = 8) (**J**). All the data are presented as mean ± SEM. Mann-Whitney U test was used for (**A, B, D–F**). The 2-tailed unpaired Student t-test was used for (**H, J**). *P < 0.05, **P < 0.01, ***P < 0.001, n.s, no significance.

Macrophage phenotypes are polarized along a continuum from pro-inflammatory, M1 phenotype to anti-inflammatory, M2 phenotype [7, 31]. In general, M1 macrophages promote atherosclerosis, while M2 macrophages improve atherosclerosis [11, 32]. Due to the importance of macrophages in atherosclerotic plaque formation [8, 11], we investigated the effect of *LXN* deficiency on macrophage phenotype, and evaluated the effects of *LXN* on oxLDL-induced inflammation and oxidative stress. *ApoE*^*-/-*^*LXN*^*+/+*^ and *ApoE*^*-/-*^*LXN*^*-/-*^ bone marrow derived-macrophages (BMDMs) were stimulated with IFN-γ and LPS to induce M1 macrophage polarization, or IL-4 to induce M2 macrophage polarization. We observed decreased expression of *iNOS* and *TNF-α* (M1 markers) in *ApoE*^*-/-*^*LXN*^*-/-*^ BMDMs under LPS conditions (Fig. 4A), while the level of *Arg1* and *CD206* (M2 markers) were elevated in *ApoE*^*-/-*^*LXN*^*-/-*^ BMDMs under IL-4 treatment (Fig. 4B). RNA sequencing further revealed that TCA cycle and fatty acid metabolism, which are considered typical metabolic characteristics of M2 macrophages [32, 33], were significantly enhanced in *LXN*-deficient BMDMs (Fig. 4C). These results suggest that *LXN* deficiency shifts macrophages toward an anti-inflammatory M2 phenotype.

Oxidative stress induced by oxLDL is an important factor in atherosclerotic plaque formation and subsequent plaque rupture [7, 34]. We were therefore interested in exploring whether the oxLDL-induced inflammatory response and oxidative damage in macrophages is abrogated by *LXN* inhibition. To this end, we incubated BMDMs from *ApoE*^*-/-*^*LXN*^*+/+*^ and *ApoE*^*-/-*^*LXN*^*-/-*^ mice with oxLDL for 48 h, and evaluated the levels of inflammatory markers and ROS production in macrophages. Decreased mRNA levels of the proinflammatory markers, *TNF-*α and *iNOS*, were apparent in *ApoE*^*-/-*^*LXN*^*-/-*^ BMDMs under oxLDL alone or in combination with INF-γ, compared with *ApoE*^*-/-*^*LXN*^*+/+*^ BMDMs (Fig. 4D). In contrast, we observed elevated mRNA levels of the anti-inflammatory M2 markers, *Arg1* and *CD206*, under oxLDL alone or combined with IL-4 in *ApoE*^*-/-*^*LXN*^*-/-*^ BMDMs (Fig. 4E). As expected, the secretion of the inflammatory markers TNF-*α* and IL-1*β* was decreased in *ApoE*^*-/-*^*LXN*^*-/-*^ BMDMs under oxLDL conditions (Fig. 4F). The production of ROS was significantly increased in oxLDL-treated *ApoE*^*-/-*^*LXN*^*+/+*^ macrophages, but was markedly lower in *ApoE*^*-/-*^*LXN*^*-/-*^ macrophages after oxLDL treatment (Fig. 4G, H). These data indicate that *LXN* deficiency promotes macrophage anti-inflammatory phenotype even under oxLDL conditions. Consistent with these data, immunohistochemical staining results showed that TNF-α, IL-6 and cleaved-Gasdermin D were significantly reduced, while Arg1 increased significantly, in atherosclerotic lesions of *ApoE*^*-/-*^*LXN*^*-/-*^ mice compared with the *ApoE*^*-/-*^*LXN*^*+/+*^ mice (Fig. 4I, J; Supplemental Fig. S6). Collectively, our data suggest that macrophage loss *LXN* is beneficial against oxLDL-induced inflammation and oxidative damage.

### Macrophage *LXN* deficiency does not affect oxLDL uptake, but increases cholesterol efflux

**Fig. 5:**
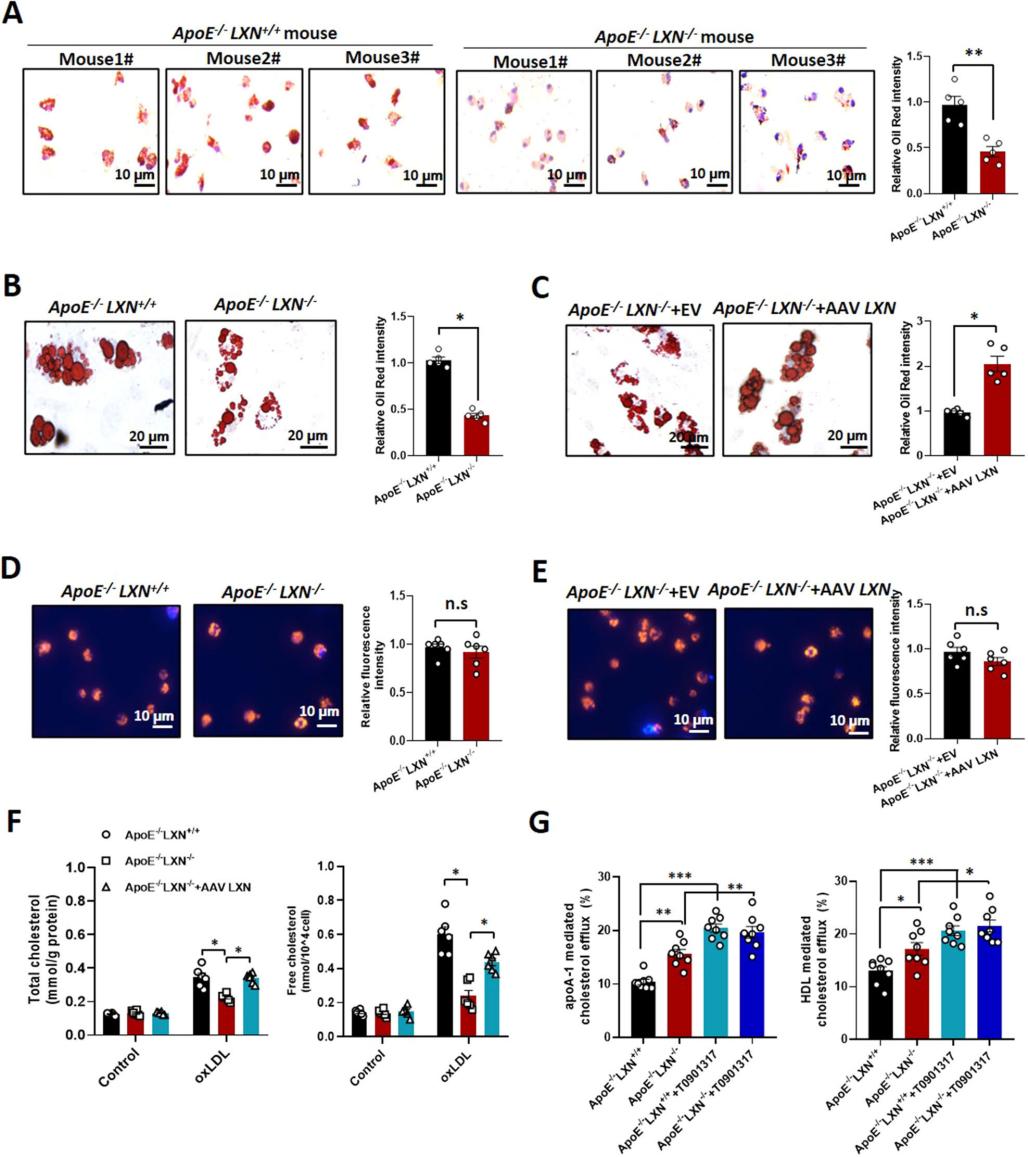
*LXN* deletion reduces macrophage foam cell formation by promoting cholesterol efflux. **A** Oil Red O-stained PMs, isolated from *ApoE*^-/-^*LXN*^+/+^ and *ApoE*^-/-^*LXN*^*-/-*^mice and treated with 20 μg/mL oxLDL for 48 h, to show foam cell formation (*n* = 5). **B** Oil Red O-stained *ApoE*^-/-^*LXN*^+/+^ and *ApoE*^-/-^*LXN*^*-/-*^BMDMs treated with 20 μg/mL oxLDL for 48 h (*n* = 5). **C** Foam cell formation in *ApoE*^-/-^*LXN*^*-/-*^ BMDMs with restored of LXN after treatment with 20 μg/mL oxLDL for 48 h (*n* = 5). **D** Uptake of Dil-oxLDL in *ApoE*^-/-^*LXN*^+/+^ and *ApoE*^-/-^*LXN*^*-/-*^ BMDMs (n = 6). **E** Uptake of Dil-oxLDL by *ApoE*^-/-^*LXN*^*-/ -*^BMDMs infected with AAV LXN (100 MOI; *n* = 6). **F** Total cholesterol and free cholesterol in WT, *LXN*^-/-^ and LXN-restored *LXN*^-/-^ BMDMs, as determined by ELISA (*n* = 6). **G** *ApoE*^*-/-*^*LXN*^*+/+*^ and *ApoE*^*-/-*^*LXN*^*-/-*^BMDMs were incubated with 1 µg/mL 3-dodecanoyl-NBD cholesterol for 4 h. Then, remove media containing 3-dodecanoyl-NBD cholesterol and gently wash the cells with PBS. After that, the cells were cultured in serum free media presence or absence of 1µmol/L LXR agonist T0901317 for 12 h. After gently wash the cells with PBS, serum-free media containing 30 μg/mL apoA-I or HDL added and incubated cells for 2 h. Culture media and cell lysate were collected, and the level of 3-dodecanoyl-NBD cholesterol in culture media and cell lysate was quantified by fluorescence spectrophotometer (*n* = 8). All the data are presented as mean ± SEM. Mann-Whitney U test was used for (**A**-**F**). One-way ANOVA was used for (**G**). *P < 0.05, **P < 0.01, n.s, no significance.

We further evaluated the effect of *LXN*-deletion on foam cell formation. Peritoneal macrophages (PMs) and BMDMs from *ApoE*^*-/-*^*LXN*^*+/+*^ and *ApoE*^*-/-*^*LXN*^*-/-*^ mice were treated with oxLDL for 48 h. *ApoE*^*-/-*^*LXN*^*-/-*^ PMs showed about 44.2% attenuation of foam cell formation (*ApoE*^*-/-*^*LXN*^*-/-*^ *versus ApoE*^*-/-*^*LXN*^*+/+*^*:*0.458 ± 0.097 *versus* 0.97 ± 0.15, *P* < 0.01) (Fig. 5A). As with the *LXN*-deficient PMs, *LXN*^*-/-*^ BMDMs also showed weak foam cell forming ability (Fig. 5B). However, reconstitution of *LXN*^*-/-*^ BMDMs with ectopic expression of *LXN* increased foam cell formation about 2fold (*P* < 0.05) (Fig. 5C). To determine whether *LXN* deficiency affects macrophage uptake of oxLDL, uptake assays of oxLDL was performed. No significant difference in oxLDL uptake was observed between wild-type and *LXN*^*-/-*^ macrophages (Fig. 5D, E). However, compared to wild-type macrophages, we observed a reduction accumulation of TC and free cholesterol in *LXN*^-/-^ macrophages; the decreased TC and free cholesterol in *LXN*^-/-^ macrophages was reversed by ectopic expression of *LXN* (Fig. 5F). Moreover, *LXN* deficiency in macrophages potently magnified apoA-1- or HDL-mediated 3-dodecanoyl-NBD cholesterol efflux (Fig. 5G), indicating that *LXN*-deficiency increased macrophage cholesterol efflux, which was further proved by the decreased cholesterol levels in the intracellular cells and cell membranes of *ApoE*^*-/-*^*LXN*^*-/-*^BMDMs using Filipin staining (Supplemental Fig. S7).

### Loss of *LXN* upregulates ABCA1 and ABCG1 in vitro and in vivo

**Fig. 6:**
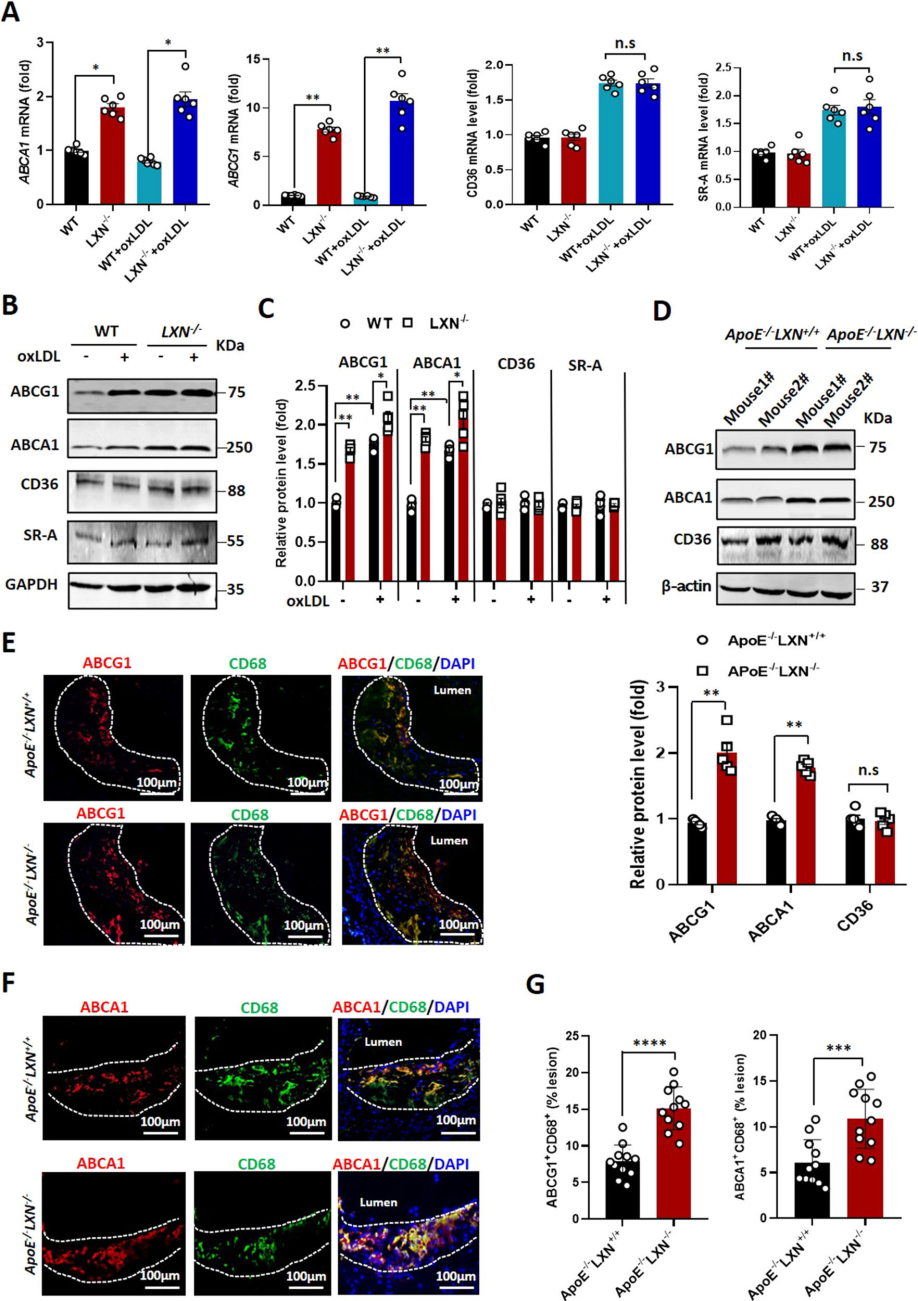
LXN regulates the expression of ABCA1 and ABCG1 in vitro and in vivo. **A** Expression of ABCA1, ABCG1, CD36 and SR-A in BMDMs isolated from WT and *LXN*^*-/-*^ mice treated with oxLDL for 48 h, as determined by qPCR (*n* = 6). **B,** **C** Expression of ABCA1, ABCG1, CD36 and SR-A BMDMs in WT and *LXN*^*-/-*^ mice treated with oxLDL for 48 h, as determined by western blot analysis (**B**) and quantification of band intensity (*n* = 5) (**C**). (**D**) Expression of ABCA1, ABCG1and CD36 in atherosclerotic plaques isolated from HFD-fed *ApoE*^-/-^*LXN*^+/+^ and *ApoE*^*-/-*^*LXN*^*-/-*^ mice, as determined by western blot analysis (*n* = 5). **E–G** Immunofluorescent staining analysis of ABCA1 (**E**) and ABCG1 (**F**) in macrophages (CD68 + ) in aortic root lesion sections from HFD-fed *ApoE*^-/-^*LXN*^+/+^ and *ApoE*^*-/-*^ *LXN*^*-/-*^ mice, and quantification of ABCG1^+^CD68^+^ and ABCA1^+^CD68^+^ areas in the aortic root lesions (*n* = 11) (**G**). All the data are presented as mean ± SEM. Mann-Whitney U test was used for (**A, C, E**). The 2-tailed unpaired Student t-test was used for (**G**). *P < 0.05, **P < 0.01, n.s, no significance.

To explore the mechanism underline *LXN* regulates cholesterol efflux, the expression of proteins related to oxLDL uptake and cholesterol efflux in *LXN*-deficient macrophage was addressed. To this end, BMDMs from wild-type and *LXN*^*-/-*^ mice were stimulated with oxLDL for 48 h, and assessed the expression of ABCA1, ABCG1, CD36 and SR-A. QPCR analysis revealed that *ABCA1* and *ABCG1* were up-regulated in *LXN*-deficient BMDMs under basal and oxLDL-treated conditions, while *CD36* and *SR-A* mRNA levels remained unchanged (Fig. 6A), and these results were confirmed by western blot (Fig. 6B, C). Consistent with these changes in ABCA1, ABCG1and CD36 in oxLDL-treated WT and *LXN*^*-/-*^BMDMs, we observed increased the expression of ABCA1 and ABCG1 in the plaques of *ApoE*^*-/-*^*LXN*^*-/-*^ mice, however there was a minimal change in CD36 levels (Fig. 6D). Remarkably, immunofluorescence analysis of atherosclerotic lesions further confirmed that *ApoE*^*-/-*^*LXN*^*-/-*^ mice possessed increased ABCG1^+^CD68^+^ and ABCA1^+^CD68^+^ areas at least 2-flod compared with *ApoE*^*-/-*^*LXN*^*+/+*^ mice (Fig. 6E–G). Together, our data demonstrate that *LXN* ablation upregulates the expression of ABCA1 and ABCG1 in macrophages in vitro or atherosclerosis in vivo.

### *LXN* deletion enhances JAK/STAT3 signaling in macrophages

**Fig. 7:**
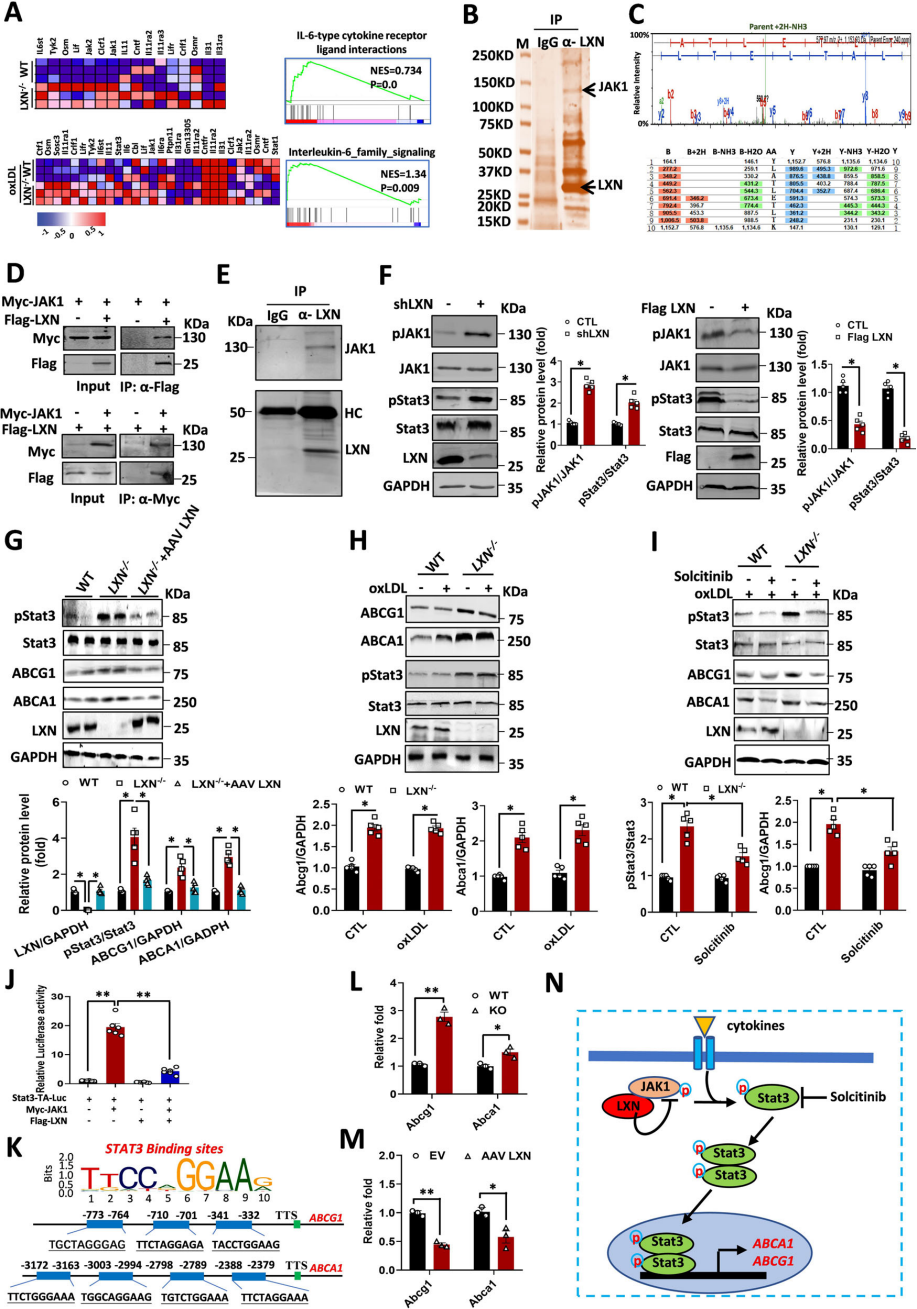
LXN inhibits STAT3 activity by targeting JAK1 in macrophages; STAT3 activation is required for up-regulation of Abca1 and Abcg1 in *LXN*-deficient macrophage. **A** Heatmap of the GSEA of representative IL-6-type cytokine receptor ligand interactions and IL-6 family signalling in WT and *LXN*^*-/-*^ BMDMs. **B,** **C** Immunoprecipitation (**B**) combined with MS/MS (**C**) to identify JAK1 as a LXN-binding protein in THP-1 cells. **D** Immunoprecipitation assay using either anti-Flag or anti-Myc antibodies with lysates from HEK293T cells 48 h after co-transfection with Flag-tagged LXN and Myc-tagged JAK1 plasmids. **E** Immunoprecipitation of endogenous LXN and JAK1 in murine BMDMs. **F** Western blot analysis of JAK1/STAT3 pathway components after knockdown or overexpression of *LXN* (*n* = 5). **G** Western blot analysis of p-STAT3, total STAT3, Abcg1 and Abca1 in WT, *LXN*^*-/-*^ BMDMs and LXN-restored *LXN*^*-/-*^ BMDMs (*n* = 5). **H** Western blot analysis of WT and *LXN*^*-/-*^ BMDMs incubated without or with 20 μg/mL oxLDL for 48 h. **I** Western blot analysis of WT and *LXN*^*-/-*^ BMDMs incubated with 20 μg/mL oxLDL in the presence or absence of 20 ng/mL solcitinib for 48 h (*n* = 5). **J** Luciferase activity in J774A.1 cells transfected with Stat3-TA-luc, Myc-JAK1 and Flag-LXN plasmids, as indicated (*n* = 6). **K** Prediction of Stat3 binding sites in *Abcg1* and *Abca1* promoter region (~3000 bp) using the JASPAR CORE database (https://jaspar.genereg.net/). **L** Representative Abcg1 and Abca1 promoter binding activity of STAT3 in WT and *LXN*^*-/-*^ BMDMs by ChIP assay (*n* = 3). **M** Representative Abcg1 and Abca1 promoter binding activity of STAT3 in BMDMs infected with AAV LXN (3). **N** Schematic diagram of the mechanism by which LXN regulates Abca1 and Abcg1 expression through the JAK1-STAT3 pathway in macrophages. The data are representative of three independent experiments, and are presented as mean ± SEM. *P < 0.05, **P < 0.01.

Next, we attempt to explore the mechanism(s) underline LXN regulates the expression of ABCA1 and ABCG1 in macrophages. Initially, RNA sequencing (RNA-seq) in wild-type and *LXN*^*-/-*^ BMDMs with or without oxLDL treatment were performed. We found that IL-6 type cytokine receptor ligand interactions and IL-6 family signaling were up-regulated in *LXN*-deficient BMDMs under basal and oxLDL treatment, respectively (Fig. 7A). We then performed immunoprecipitation assays to determine the targets of LXN, and confirmed that JAK1 is a partner for LXN in THP-1 macrophages (Fig. 7B, C). These data are suggestive of a crosstalk between LXN and the IL-6/JAK/STAT signal pathway in macrophages. To verify the interaction between LXN and JAK1, Flag-tagged LXN and Myc-tagged JAK1 plasmids were co-transfected into HEK293T cells. We found that immunoprecipitation with anti-Flag resulted in co-precipitation of Myc-JAK1. However, in the absence of Flag-LXN, the anti-Flag antibody did not precipitate Myc-JAK1 (Fig. 7D). The interaction between endogenous LXN and JAK1 was further validated through co-immunoprecipitation in BMDMs (Fig. 7E). Functionally, we demonstrate that inhibiting LXN by shLXN dramatically increases the phosphorylation of JAK1 and STAT3 in J774A.1 cells, and overexpression of LXN does indeed inhibit JAK1/STAT3 phosphorylation (Fig. 7F). Together, our data demonstrate that LXN inhibits JAK1/STAT3 activity in macrophages.

### The activation of JAK1/STAT3 is required for the upregulation of ABCA1 and ABCG1 in *LXN*-deficient macrophages

It is well know that ATP-binding cassette transporters are crucial for cholesterol efflux [16, 17], and STAT3 has been reported to regulate ATP-binding cassette transporter expression [35, 36]. We therefore examined whether the JAK1/STAT3 pathway is enhanced in *LXN*^*-/-*^ macrophages. As expected, we observed significantly increased STAT3 activation (p-STAT3) and expression of ABCG1 and ABCA1 in *LXN*^*-/-*^ BMDMs compared with WT BMDMs. Importantly, this increase was dramatically reversed by overexpression of LXN (Fig. 7G). We confirmed that STAT3 phosphorylation, ABCG1 and ABCA1 were dramatically increased in oxLDL treated *LXN*^*-/-*^ BMDMs, in comparison to wild-type BMDMs (Fig. 7H). This increase of p-STAT3, ABCG1 and ABCA1 in *LXN*^*-/-*^ BMDMs was significantly abrogated in the presence of a JAK1 specific inhibitors solcitinib (Fig. 7I). In addition, we isolated peritoneal macrophages (PMs) from *LXN*-deficient mice, and treated with oxLDL to induce foam cell formation. We found that *LXN* deficiency showed an attenuation of foam cell forming ability, and treatment of *LXN*-deficient macrophage with JAK inhibitor solcitinib could reverse this inhibitory effect (Supplemental Fig. S8). These results indicate that inhibiting JAk1 in macrophages would significantly inhibit the effect of *LXN*-deficiency on macrophage function related to atherosclerosis. To clarify whether LXN can affect the DNA binding ability of STAT3, we performed a luciferase (Stat3-TA-Luc) assay in J774A.1 cells. The results showed that ectopic expression of JAK1 markedly increased Stat3-TA-luc activity, however, this activity was significantly suppressed when co-expressing JAK1 and LXN (Fig. 7J), suggesting that LXN inhibits JAK1-mediated DNA binding activation of STAT3. Further analysis was conducted using the Jaspar online tool to identify approximately ~3000 bp upstream transcription factor binding sites of the TSS site in mouse *ABCG1* and *ABCA1* genes [37], and the putative STAT3 binding sites were predicted with an 80% relative profile score (Fig. 7K, Supplemental Tables S1 and S2). ChIP analysis confirmed that LXN has an inhibitory effect on STAT3 binding activity with *ABCG1* and *ABCA1* promoter regions in wild-type and *LXN*^*-/-*^ BMDMs (Fig. 7, L and M). Collectively, our data confirm the existence of a macrophage LXN/JAK1/STAT3 signalling axis, which regulates of ATP-binding cassette transporters, suggesting that activation of JAK1/STAT3 signalling is required for the expression of ABCG1 and ABCA1 in *LXN*-deficient macrophages (Fig. 7N).

### Exogenous inhibition of LXN attenuates atherosclerosis in *ApoE*^*-/-*^ mice

**Fig. 8:**
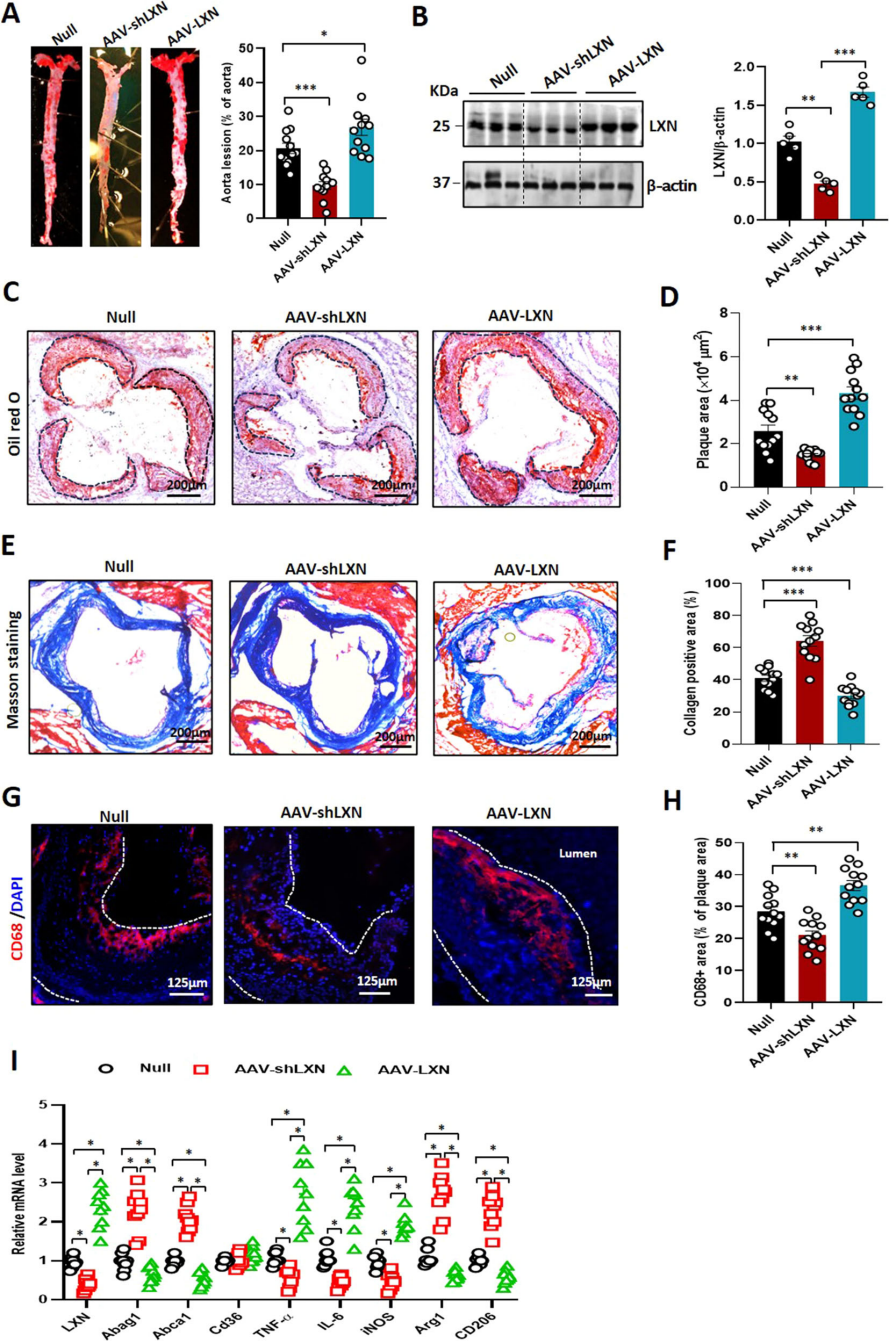
LXN inhibition attenuates and overexpression accelerates atherosclerosis in *ApoE*^-/-^ mice. *ApoE*^-/-^ mice treated with adeno-associated virus to silence LXN or overexpress LXN were fed a HFD for 12 weeks before analysis. **A** Representative images of *en face* Oil Red O-stained aortas from each group (n = 12). **B** Expression of LXN in aortas from each mouse from each group, as assessed and quantified by western blot analysis (*n* = 5). **C,** **D** Representative images of Oil Red O-stained cross-sections of aortic roots from each group (**C**), and quantification of plaque area (*n* = 12) (**D**). **E,** **F** Representative images of collagen (blue) content from each group (**E**) and quantification of collagen content (*n* = 12) (**F**). **G,** **H** Representative images of macrophage infiltration in murine atherosclerotic lesions by immunofluorescence staining of CD68 (**G**) and quantification of CD68^+^ macrophage area in each group (*n* = 12) (**H**). **I** mRNA levels of genes in atherosclerotic lesions, as determined by quantitative real-time PCR (*n* = 9). All the data are presented as mean ± SEM. One-way ANOVA was used for statistical analysis. *P < 0.05, **P < 0.01, ***P < 0.001.

Finally, we addressed whether exogenous changes in LXN expression can have therapeutic effects in atherosclerosis. To this end, we generated adeno-associated virus to deliver the *LXN* gene (AAV-LXN) or LXN-depleting shRNA (AAV-shLXN) into HFD-diet *ApoE*^-/-^ mice. *En face* Oil Red O staining showed that the area of atherosclerotic lesions in mice treated with AAV-shLXN was significantly reduced, on the contrary the area of atherosclerotic lesions in mice treated with AAV-LXN was significantly decreased (Fig. 8A). As expected, LXN was decreased in AAV-shLXN-treated mice aorta and increased in AAV-LXN-infected mice (Fig. 8B). Histological evaluation revealed that overexpression of LXN exacerbated the formation of atherosclerotic plaques (Fig. 8C, D) and significantly weakened the stability of plaques, whereas *LXN*-knockdown had the opposite effect (Fig. 8E, F). Further analysis of the atherosclerotic plaques revealed an increase in CD68^+^ macrophage infiltration in AAV-LXN-infected mice, whereas macrophage infiltration was attenuated in AAV-shLXN-treated, HFD-diet *ApoE*^-/-^ mice (Fig. 8G, H). Finally, qPCR analysis of atherosclerotic plaques further indicated that, in HFD-fed *ApoE*^-/-^ mice, *LXN* knockdown by AAV-shLXN-treated resulted in a marked increases in mRNA levels of *ABCG1*, *ABCA1*, *Arg1* and *CD206*. Meanwhile, pro-inflammatory genes, such as *IL-6* and *TNF-α*, were dramatically up-regulated in atherosclerotic plaques when mice were infected with AAV-LXN (Fig. 8I). Taken together, these findings confirm that suppressing LXN by genetic disruption can effectively improve atherosclerosis in *ApoE*^-/-^ mice.

## Discussion

Macrophages are central to the pathogenesis of atherosclerosis [7, 8, 34]. LXN is abundant in macrophages and plays critical roles in inflammation [26, 28, 29], however, the role of LXN in atherosclerosis has not been clarified. This study shows that *LXN* deficiency markedly improves atherosclerosis through the inhibition of foam cell formation. We first demonstrated that LXN expression is increased and colocalizes with macrophages in both human and murine atherosclerotic lesions, and that *LXN* deficiency decreases atherosclerosis in *ApoE*^*-/-*^ mice. These findings are supportive of a key function of LXN in macrophages in atherosclerosis. Experiments in vivo and in vitro further demonstrate that *LXN*-deficient macrophages possess enhanced anti-inflammatory and antioxidant phenotypes. LXN directly targets and inhibits JAK1, which attenuates the activity of STAT3, consistent with our previous reports on colorectal inflammation and colorectal cancer in *LXN* knockout mice [26, 27]. *LXN*-deficient macrophages enhance cholesterol efflux by activating the STAT3/ATP-binding cassette transporter pathway, thus inhibiting the formation of foam cells and atherosclerosis. Importantly, adeno-associated virus mediated gene therapy to reduce LXN in *ApoE*^*-/-*^ mice revealed that *LXN* deficiency improves atherosclerotic plaque formation in the mouse atherosclerosis model. Thus, LXN may be a new therapeutic target for anti-atherosclerosis.

Vascular endothelial injury and subsequent monocyte infiltration are the key initiation steps of atherosclerosis [38, 39]. Monocytes attach to the inflamed vascular endothelium and infiltrate into the intima of the artery, where they differentiate into macrophages and transform into foam cells [2, 11]. We previously reported that LXN in endothelial cells is critical for vascular homeostasis [24], and plasma LXN level is elevated in patient with coronary heart disease [40]. In this study, we further show that LXN is enriched in atherosclerotic plaques with the infiltration of macrophages in the tunica intima during atherosclerosis, therefore thus, suggesting the physiological role of macrophage-derived LXN in atherosclerotic plaque formation. We also verified the critical function of macrophage-derived LXN in atherosclerosis by BMT experiments.

Macrophages in plaques are critical for the development of atherosclerosis [7, 8], and these cells are divided into two groups: classically activated (M1) and alternatively activated (M2) macrophages [33, 41]. M1 macrophages are associated with host defence and produce pro-inflammatory cytokines such as TNF and IL-1β, while M2 macrophages are associated with tissue repair, wound healing and metabolic processes, maintaining tissue homeostasis [7, 8, 10–12]. Accordingly, our results confirm that *LXN* deficiency promotes M2 macrophage polarization, protecting them against oxLDL-induced oxidative damage. Consistent with this, RNA-seq analysis further verified that *LXN* loss enhances citrate cycle and fatty acid metabolism in macrophages, both of which are characteristic features of the M2 phenotype [33]. Additionally, the disruption of lipid homoeostasis is a main contributor to foam cell formation [14, 42]. Here, we also demonstrate that *LXN* deletion in macrophages inhibits oxLDL-induced foam cell formation, without affecting oxLDL uptake, but accelerating cholesterol efflux.

It is well known that Gasdermin D (GsdmD) is the final executor of inflammasome activity, which involved in the release of inflammatory factors such as IL-1β, and mediating cell pyroptosis [43, 44]. Recent studies have shown that GsdmD -mediated pyroptosis is involved in the initiation, progression, and complications of atherosclerosis that involve the endothelial cells, macrophages, and smooth muscle cells [45]. In particular, it has been reported that cleaved-Gasdermin D is present in human and mouse atherosclerotic plaques [46]. Moreover, there is evidence to suggest that GsdmD mediates inflammation-induced defects in reverse cholesterol transport and promotes atherosclerosis. *GSDMD*^−/−^ mice exhibit decreased atherosclerotic lesion area [47, 48]. One of the important mechanisms of GsdmD affecting the progression of atherosclerotic plaque may be that *GsdmD* deficiency blocks the release of IL-1β and IL-18 from inflammatory immune cells [49, 50]. In this study, we found that compared to ApoE^-/-^ mice, inflammatory factors IL-1β in plaques of ApoE^-/-^LXN^-/-^ mice was decreased significantly (Fig. 2J). Consistent with this, the plasma IL-1β levels were lower in ApoE^-/-^ LXN^-/-^ mice fed with HFD (Supplemental Fig. S4), and the secretion of IL-1*β* was decreased in ApoE^-/-^LXN^-/-^ BMDMs under oxLDL conditions (Fig. 4F). These results indicate that *LXN* deficiency can indeed suppress the level of IL-1*β* both in atherosclerotic plaques and BMDMs. We also detected the expression of cleaved-GsdmD in atherosclerotic plaque (Supplemental Fig. S6). Interestingly, immunohistochemistry showed that *LXN* deletion reduced the level of cleaved-GsdmD in plaque while reducing the plaque area (Fig. 2E; Supplemental Fig. S6), suggesting that LXN may participate in GsdmD mediated inflammasome activity, but its mechanism needs to be further clarified in the future.

As is well known, the STAT3 pathway has anti-inflammatory functions in macrophages [51, 52]. Multiple studies have shown that the STAT3 pathway mediates polarization of M1/M2 macrophages, as well as the expression of ATP-binding cassette transporters [53, 54]. Here, our results show that *LXN* loss in murine BMDMs markedly increases IL-6/STAT3 signalling and the downstream expression of ATP-binding cassette transporters. Indeed, Frisdal et al. reported that lipid homeostasis disruption in macrophages leads to cholesterol accumulation, which is accompanied by an increase in IL-6 secretion. On the other hand, extracellular IL-6 promotes ABCA1-mediated free cholesterol efflux by activating the Jak-2/Stat3 signaling pathway, reducing lipid accumulation in macrophage, indicating the negative feedback regulation of IL-6 on lipid accumulation [53]. In our study, although the transcriptome results of BMDM cells suggest that IL-6 type cytokine receptor ligand interactions and IL-6 family signaling (such as IL-6/STAT3 pathway) were up-regulated in *LXN*-deficient BMDMs under basal and oxLDL treatment. However, in our study, we found that inflammatory factors, such as TNF-α, IL-1β, MCP-1 and IL-6, were decreased significantly in plaques in *ApoE*^*-/-*^*LXN*^*-/-*^ mice; Immunohistochemical staining also showed that TNF-α and IL-6 were significantly reduced, while Arg1 level was increased significantly, in the atherosclerotic lesions of *ApoE*^*-/-*^*LXN*^*-/-*^ mice compared with the *ApoE*^*-/-*^*LXN*^*+/+*^ mice, indicating that the activation of STAT3 and subsequent expression of ATP-binding cassette transporters in *LXN* knockout BMDMs and atherosclerotic plaques in *ApoE*^*-/-*^*LXN*^*-/-*^ mice were not caused by increase of IL-6. We further demonstrated that LXN directly interacts with JAK1, an important member of the IL-6/STAT3 signaling pathway, in macrophages. Importantly, this interaction inhibits the activity of JAK1 and STAT3, while *LXN* deficiency can enhance the JAK1/STAT3 signaling pathway and ultimately increase the expression of ABCA1 and ABCG1, and the increase of ABCA1 and ABCG1 in *LXN*^*-/-*^ macrophages was reversed by ectopic expression of LXN or treatment with a STAT3 inhibitor, as expected. Therefore, our study emphasizes that macrophage LXN directly regulates JAK1/STAT3 activity through its interaction with JAK1, thereby regulating ATP-binding cassette transporter, which has not been previously reported.

Finally, we tested whether disrupting the *LXN* gene in HFD-diet *ApoE*^*-/-*^ mice would yield therapeutic effects. Increased LXN expression substantially exacerbated atherosclerosis, while *LXN* disruption markedly reversed atherosclerosis, including decreased macrophage infiltration and reduced macrophage-rich fatty streaks. Therefore, our study provides the experimental evidence that *LXN* targeted delivery could intervene atherosclerosis, which may open new therapeutic strategies for preventing atherosclerosis and cardiovascular disease.

In conclusion, the present study demonstrates that LXN plays previously unrecognized pathophysiological roles in atherogenesis. LXN levels are increased in atherosclerotic lesions and colocalize with macrophages. LXN is a negative regulator of the macrophage anti-inflammatory phenotype and cholesterol efflux; thus, LXN inhibition may play a braking role in the formation of foam cell and atherosclerosis. Our findings provide new clues for the physiological role of macrophage LXN in the regulation of atherosclerosis and identify macrophage LXN as a target to prevent cardiovascular disease.

## Materials and Methods

### Cell lines and reagents

THP-1 cells purchased from ATCC were cultured in DMEM with 5% CO2 at 37 °C. Mouse monocyte macrophages J774A.1 cells purchased from Procell Life Science &Technology Co., Ltd. (Wuhan, China) were cultured in RPMI 1640 medium. The following antibodies were used: mouse anti-LXN (Sino Biological Inc, 1:1000 for WB), rabbit anti-LXN (abcam, 1:1000 for WB, 1:100 for IF), mouse anti-human CD68 antibody (eBioscience, 1:100 for IF), rat-anti mouse CD68 antibody (eBioscience, 1:100 for IF), rabbit anti-αSMA (Cell Signaling Technology™, CST, 1:100 for IF), anti-CD36 (Cell Signaling Technology™, CST, 1:1000 for WB), anti-SR-A (Cell Signaling Technology™, CST, 1:1000 for WB), anti-Abcg1 (Cell Signaling Technology™, CST), anti- cleaved-Gasdermin D (Cell Signaling Technology™, CST, 1:100 for IH), anti-Abca1 (Cell Signaling Technology™, CST, 1:1000 for WB, 1:100 for IF), anti-STAT3 (eBioscience, 1:1000 for WB) and anti-pY705-STAT3 (Beyotime, 1:1000 for WB), F4/80 eFluor 450 (eBioscience, 0.5 µg/test for Flow cytometric), CD16/32 PerCP-Cyanine5.5 (eBioscience, 0.125 µg/test for Flow cytometric), CD206 APC (eBioscience, 0.25 µg/test for Flow cytometric). FITC-conjugated secondary antibodies (1:250 dilution) and TRITC-conjugated secondary antibodies (1:300 dilution) were purchased from Invitrogen. *LXN* overexpressing, silencing and null adeno-associated virus were constructed by Sangon Biotech (Shanghai, China). Mouse serum TC, LDL-C and TG ELISA Kits were purchased from Nanjing Jiancheng Bioengineering Institute (Nanjing, China). ELISA Kits for mouse serum TNF-a, IL-1β, and IL-10 were purchased from Beijing Solaybao Technology Co., Ltd (Beijing, China).

### Human atherosclerotic tissues

The use of human samples in this study was approved by the Ethics Review Committee of Dongguan People’s Hospital (KYKT2021-022-A), and a signed informed consent form was jointly obtained from the patients. Paraffin embedded specimens of human aorta with atherosclerotic plaques were acquired from Guilin Medical College and Dongguan People’s Hospital.

### Animals and animal atherosclerosis studies

All animal experiments were performed under the approval of the Guide for the Care and Use of Laboratory Animals and were endorsed by the Animal Ethics Committee of Guangxi Normal University (approval number: GXNU2019-013). *LXN*^-/-^ mice were purchased from the RIKEN BioResource center (Japan) [22, 26]. *ApoE*^*-/-*^ mice (C57BL/6 background) were purchased from GemPharmatech Co., Ltd (Nanjing, China). *LXN*^*-/-*^ mice were inter-crossed with *ApoE*^*-/-*^ mice to generate *ApoE*^*-/-*^*LXN*^*-/-*^ mice [24], Mice genotype was determined by PCR using the primers as below:

Pro-F, 5’-CGTTAGACTTTAAAATGCTCACTTTGGAAGCCCATACT-3’; Lax-R, 5’-CCTCCTTGCTGGCCTGCTGGACCGTCTGCACC-3’; Lxn-wt-TF2, 5’-AATCTGTACGTGAAACAGCCAGC-3’; Lxn-PGK-TR2, 5’- ATTTGTCACGTCCTGCACGAC-3’; Apoe-del82-tF1: 5’-TGCCTAGTCTCGGCTCTGAACTAC-3’; Apoe-del82-tR1: 5’-CAACCTGGGCTACACACTAATTGAG-3’. Mice (*ApoE*^*-/-*^ *LXN*^*+/+*^ and *ApoE*^*-/-*^*LXN*^*-/-*^) fed a HFD (D12108C, Research Diets, Inc. NJ, USA) for 12 weeks were sacrificed using carbon dioxide (CO_2_) in accordance with the NIH Guidelines for the euthanasia of animals. Plasma was prepared by centrifugation of whole blood at 8000 rpm for 5 min at 4 °C. The entire aorta was removed and opened longitudinally from the aortic root to the iliac bifurcation for *en face* staining with Oil Red O, and analyzed using a digital camera (Nikon D70) Image Pro software.

For aortic sinus analysis, the whole heart and aorta were dissected, and serial 6-μm thick cross-sections of the aortic sinus were made using a cryotome. In detail, from the first cross-section in which the leaflets of the aortic valves appeared upward, continuous sectioning is performed, spanning 800 µm of the proximal aorta, and sections are stained with hematoxylin and eosin (H&E), and imaged using an Invitrogen EVOS FL Auto2 microscope (Thermo Fisher Scientific). The frozen sections of the aortic root were stained using Oil Red O staining kit (Solarbio, Beijing, China) according to the manufacturer’s instructions. Morphological analysis of the collagen contents in the lesion was performed by staining with a Masson’s trichrome staining Kit (Solarbio, Beijing, China). For each mouse and staining, 6-9 root sections were analyzed and data were averaged, with 8 mice in each group.

BMDMs were isolated from femurs and tibias of 6-week-old *ApoE*^*-/-*^ *LXN*^*+/+*^ and *ApoE*^*-/-*^*LXN*^*-/-*^ mice, and cultured in RPMI-1640 medium (supplemented with 10% FBS, and 50 ng/mL murine M-CSF). Mouse peritoneal macrophages from *ApoE*^*-/-*^*LXN*^*+/+*^ and *ApoE*^*-/-*^*LXN*^*-/-*^ mice were isolated 3 days after intraperitoneal injection with a 4% thioglycolate solution. Macrophages were selected by fluorescence-activated cell sorting of F4/80^+^ (F4/80, eFluor 450, eBioscience) and CD11b+ cells (CD11b, PE-Cyanine7, eBioscience).

For bone marrow transplantation, bone marrow was collected from six-week-old, sex-matched donor mouse femurs and tibiae. Recipient mice were exposed to lethal irradiation with two doses of 5.5 Gy (total 11 Gy), at a 4 h intervals to minimize radiation toxicity, and then transplanted with 10^7^ bone marrow cells by tail vein injection. After 4 of weeks recovery, the transplanted mice were fed a high-fat diet (HFD) for 12 weeks.

### Production of plasmid, Small interfering RNA and recombinant AAV vectors

To construct LXN expression plasmid, LXN cDNA was amplified by PCR using the primers containing BamHI and EcoRI sites. LXN was sub-cloned into pFlag-CMV vector as described in our previously published paper [22]. AAV-LXN and AAV-siLXN were produced by pAAV2/9 vectors (Addgene, Plasmid #112865). Briefly, the sequence of LXN cDNA and siLXN were subcloned into AAV serotype 2/9 vectors to construct the AAV-LXN and AAV-siLXN expression vectors, respectively. Infectious AAV2/9n vector particles were generated in HEK293T cells, using a dual-plasmid co-transfection system for packaging. The transfected cells were harvested and were lysed by repeated freeze-thaw cycles 48 h after transfection. For gene therapy, *ApoE*^*-/-*^ mice fed a HFD for 12 weeks, and AAV (2.5 × 10^9^ pfu) in 50 μL sterilized PBS was injected weekly into the tail vein. Mice were killed for macrophage isolation and quantification of atherosclerotic lesions.

### Immunohistochemistry and immunofluorescence staining

Cross-sections of the aortic root were stained with primary antibodies, followed by HRP-conjugated secondary antibodies, and developed with DAB substrate. Frozen sections were fixed, and processed with antibodies according to standard procedures. Images were captured using a Zeiss LSM 510 Meta inverted Confocal Microscope.

### Transcription factor (TF) binding site prediction

The murine Abcg1 (Gene ID: 11307) and Abca1 (gene ID:11303) sequences were download from the NCBI database. The promoter sequence, about 3000 bp upstream of the TSS, was extracted using the NCBI Graphical Sequence Viewer (v3.44.1). TF sites were predicted using the online tool, Jaspar (https://jaspar.genereg.net/). The relative profile score threshold was 80%; the results are provided in Supplementary Tables S1 and S2.

### ChIP

ChIP was performed using the Simple ChIP Plus Enzymatic Chromatin IP Kit (Magnetic Beads #9005), according to the manufacturer’s instructions as reported previously [55]. PCR was performed in a FX96TM Real-Time system (Bio-Rad) using the iQ SYBR Green supermix (Bio-Rad). The primer sequences used were as follows: *Abcg1* promoters, F:5’-TCACCGCACACTAGCGCTAA-3, R:5’- AATGGGATAGGGAAGAAGGCA-3’; *Abca1* promoters, F 5’-AAGAACTTTTCCTCTAGTCT-3, R:5’-TTGGGATGAGACGAAAAACT-3’.

### Foam cell formation

Cells were incubated with oxLDL (Unionbiol, Beijing, China) for 48 h, after which the cells were fixed with 4% formaldehyde, stained with Oil Red O (Solarbio, Beijing, China).

### Uptake of oxLDL

BMDMs were incubated with 10 μg/mL fluorescence-labeled oxLDL (Dil-oxDL, Unionbiol, Beijing, China) for 4 h at 37 °C to assess uptake of Dil-oxLDL. Fluorescence intensity was analysed under a fluorescence microscope and quantified with Image Pro Plus Software.

### Filipin staining of cell cholesterol

*ApoE*^*-/-*^*LXN*^*+/+*^ and *ApoE*^*-/-*^*LXN*^*-/-*^BMDMs cultured in six-well plate and treated with 20 μg/mL ox‑LDL for 48 h. The cells were washed with PBS for 3 times and then stained with Filipin staining kit according to the manufacturer’s instructions (GENMED, Shanghai, China).

### Cholesterol efflux assay

*ApoE*^*-/-*^*LXN*^*+/+*^ and *ApoE*^*-/-*^*LXN*^*-/-*^BMDMs were incubated with 1 µg/mL 3-dodecanoyl-NBD cholesterol (Cayman Chemical) for 4 h. After that, remove media containing 3-dodecanoyl-NBD cholesterol, and gently wash the cells 3 times with PBS. Afterwards, the cells were cultured in serum free media presence or absence of 1µmol/L LXR agonist T0901317 (MCE®, Shanghai, China) for 12 h. Gently wash the cells with PBS, then add serum-free media containing 30 μg/mL apoA-I (Unionbiol, Beijing, China) or HDL (Unionbiol, Beijing, China) for 2 h. Collect culture media and cell lysate, respectively. The level of 3-dodecanoyl-NBD cholesterol in culture media and cell lysate was quantified by fluorescence spectrophotometer. The rate of cholesterol efflux is expressed as a proportion of cholesterol moved from cells to the acceptor. The following formula is used: %Cholesterol Efflux=Media 3-dodecanoyl-NBD cholesterol/(Media 3-dodecanoyl-NBD cholesterol+ Cell lysate3-dodecanoyl-NBD cholesterol)×100%.

### ROS analysis

Wild-type and *LXN*^*-/-*^BMDMs were stimulated with oxLDL for 48 h. ROS were determined using a DCFH-DA kit (Solarbio, Beijing, China) according to the manufacturer’s instructions.

### RT-qPCR assay

Total RNA was extracted and purified using TRIzol Reagent (Life Technologies, Rockville, MD) and RNeasy Mini kit (Qiagen Inc). qRT-PCR was performed using a cDNA Synthesis kit and SYBR Green Master Mix Kit (Exqion), and utilizing the primers detailed in Table S3.

### RNA-Seq

Total RNA was isolated from BMDMs using TRIzol reagent (Life Technologies, Rockville, MD). RNA integrity was assessed using the RNA Nano 6000 Assay Kit of the Bioanalyzer 2100 system (Agilent Technologies, CA, USA). The purified RNA samples were sequenced at Novogene corporation (Beijing, China) as reported previously [22].

### MS/MS analysis

THP-1 cells were lysed and immunoprecipitated using anti-LXN antibody. Protein complexes were assessed by SDS-PAGE. Gel slices were excised and digested with trypsin. Tryptic peptides were analysed using a MALDI-TOF/TOF 5800 mass spectrometer (AB SCIEX, USA) as reported previously [56].

### Luciferase assay

J774A.1 cells were transfected with pGL3-renilla plasmid or pGL3-mCD36 promoter with and without indicated mutations using lipofectamine transfection reagent as described with little modification [57, 58]. After transfection for 24 h cells were lysed, and the luciferase and renilla activity were assayed and expressed as relative luciferase units (RLU).

### Western blot

Cells or tissues were lysed using RIPA buffer, and quantified using a BCA protein assay kit (Pierce Biotechnology, Rockford, Illinois, USA). Western blot was performed as reported previously [56].

### Statistical analysis

For statistical analysis, GraphPad Prism 8.0.1 was used. Data are presented as mean ± SEM. Normality and equivariance tests were performed, two-tailed, unpaired Student’s t-tests was used if the data passes tests; For abnormally distributed data, Mann-Whitney U test was used; Comparisons among three or more groups were performed using one-way ANOVA. For all statistical tests, P < 0.05 was considered statistically significant (*P < 0.05, **P < 0.01, ***P < 0.001, ****P < 0.0001).

## Supplementary information

Supplemental data

## Data Availability

Materials, experiment procedures, data collection protocols, and analytic methods will be made available to other researchers as requested for purposes of experiment reproduction, procedural replication, and for collaborative study.
