# Supplementary material for: Latexin deficiency limits foam cell formation and ameliorates atherosclerosis by promoting macrophage phenotype differentiation
Source: Cell Death Dis. 2024 Oct 18;15(10):754. doi: 10.1038/s41419-024-07141-3 (PMC11492231; doi:10.1038/s41419-024-07141-3)

**Data Supplement**

***Latexin* Deficiency Limits Foam Cell Formation and Ameliorates Atherosclerosis by Promoting Macrophage Phenotype Differentiation**

He G., et al.

**Prediction of transcription factor binding sites of mouse ABCG1 and ABCA1.**

**>Mouse Abcg1 Promoter (-3147-1) (mouse Abcg1, Gene ID: 11307)**

TCTAAGTGCTACTGCCATTGAAATGATGCCTGCATCTTATTAGAAATGATTGTAACATGGTAGAAGTAAGTCAGAGACTGTTCAGGGTATACATCCTGTAGGAGGGGATGGGTATGTTATAAGCAAACCTTGCATCCTTGCAAGAGATATGAGGTTTTGGCATTGGATAGGGGTCTGGAAACCAGACTCCCGAAGGTATCAAGGATGACAGTATTAATGGAAAGCCTCCTTGCTGAAACAGGAAGTGGCTTTCCTTCCAAAAGTCCTCAAGGCCAACTAGGTCCATCTATGTATGGTCGTGTCTTGGCAGAAATTCCCCCTGGGGTTGTGTCTGGCCTGGACAATGCTGAGCTGTGCCTGCTTCACATTGACTGGCCTGGTACTTGTGGTTAGAAGGTCTCTGCCAAGTGGGTTGTAGCTCCCTCTGTAGTGCCTTGGGACTCAGCTACTTGATTTTTCCTTCCATGTGACTCAGCCTGGGAGGGACCATCAGGACTAGGCACCATTAACTGGGTGCAGTTGATAGGATGGAAGAGCAATAGTCTTACTTAGAGTTTCTCTTGCTGCAACAAAACACCATCGCCAAAAAGCTAGTTGAAAAGGAAAGGGTTAATTCAGCTTACACTTCTAAATAGCTGTTCATCACCAAAGGAACTTAAAGGTGGCAGGAACCTGGAGGTAAGAACTGATGTAGAGGCCATGGAGGGTGCTGCTTACTGGTTTGCTTCCCCTGGCTTGCTCAGCCTGCTTTCTTATAGAACCCAGGACCACCAGCCCAGGGGTGGAAACACTCATAATGGGCTGGGCCCTCCCCATCAATCACTAATTAAGAAAATGCCTTATAGCCAGATCTTATGGAGGCATTTTCTCAGTTAAGGTCCCCTCTTTTTAGATGACTCTAGTTTGTGTGTCAAATTGACACAAGACTAGCCAGCTTAGAAGGTAACAGTCCCTGAGAGAAACTACCACATGAAGGCAGAGTACTGAGAAATCAAAGTGTGTGTGTGCATGTGTGTGTACTTATCCTTGGCCCTGTTGCACATGAAACCAGTGTTCAGAACTTACTCACTACAGAATGATTGGAACCTACTCACTGCAGAAAACAGTGTACTTGGCCAGTCAAAACTGGCTTTCTGTCACTTACAGCACAAGTCTGTCCAACTCTGCCACTCTCTCATTCCCTGCTCAGGCATGAAGGGGATGCCAGGAGTAAACAATGGAAAAGATACTTGTTTGTAGGTAGGAAGCACCCATCACTTGTGGGGTCCATTGTGGCCAGGGGGACTACTACCCCATGGACACATATATGAGTGGGAACCCTTTCAGTGGCCCAAGCCACACCAGTGAGAACCCCACACATGGCTGTCTCTGTCTGAGCCCTGTGCATTCTCAGGAACCCAGAAAGCCCTGCCTCCCATCTGAAGCTCCAGTACACAGGCTGCATGGAGGCTGCTGGGGGCTGGAAGCTCATTGGCCTACTTTTGAATTAAAGGTTCATCAAAGGAATGTGAATCAAAGGGGGCGTGGAAATGGGGGAAGGGGTGGGGATGGGGTTGAGTAGAAGGGGCTTGAGAAAGAGAGAAATGCCAGAAGAAAATTCTGAAAGTAGGGAGAACAGACAGAGGGTAGATATCTGCAAAACCAGGCATGCTGATACCATGGCCTGTGTCCTGGTCACATTATTCAGTGTCTCCCCTTGTCCATTCTTGCTGCTTTACAGCATGGTTGGGCACCAGGGCAGACAGTAGCCCAGGCCCCAGAAACCTTTCTCACTCCCTGGGAGGTTTGTACTCTTTTTTTTTTTAATCTGAATTTTATTTTTATTTTTTTTCTATCCTTTTTTTTTCCATTTTTTATTAGGTATTTAGCTCATTTACATTTCCAATGCTATACCAAAAGTCCCCCATACCCACCCACCCCCACTCCCTTACCCACCCACTCCCCCTTTTTGGCCATGGTGTTCCCCTGTACTGGGGCATATAAAGTTTGCGTGTCCAATGGGCCTCTCTTTCCAGTGATGGCCGACTAGGCCATCTTTTGATACATATGCAGCTAGAGTCAAGAGCTCCGGGGTACTGGTTAGTTCATAATGTTGTTCCACCTATAGGGTTGCAGATCCCTTTAGCTCCTTGGGTACTTTCTCTAGCTCCTCCATTGGGAGCCCTGTGATCCATCCATTAGCTGACTGTGAGCATCCACTTCTGTGTTTGCTAGGCCCCAGCATAGTCTCACAAGGAGGTTTGTACTCATAACCTTCTAGAATGCCCTGGCTTTGTCAGCCTTCTTGATACCAGATAGGGCCATCGGTCCCTCCGGTATTAGAAGCCCACCCTCTTCCCTCCAAACTCAGCCACACCCACTTTTCTTTCTGTGA**TGCTAGGGAG**AAGCTTAGCTGAGGTCAAGGATCTGAGGATGTGACAACCACTTGCCTGCCCCT**TTCTAGGAGA**GCAGAAAGTACCCCAGCACACCCAAGTGTTGGAGCGGGTGGCTCTACTGTGCTGGGGGTGACAGAGAATCATTTCAACAGAAGACTTAGTTTAGGATCTGAGGTTGGGATTAAAGATTTTCACCACGGTTACCACGCAACGTCCCATTTACAAATCACAGGGGTTTCAGTAGGGTACAAGAACGTGGCTGTGTAAGACACTGGAAACACCCCTGTGGCTTATTTGGGGATGGAGTGGTGCTGATGGAGACCTAGGAGCTATCTGTATGAACGCCCATGCTGCCTCAATTTAGGGTCACCGCACACTAGCGCTAAGGGAAAAGCCCTGGGGCTGTTTTCTACGTTTGTCCCTGGGGCTTC**TACCTGGAAG**GCATGCATAACAAAGGCAGAACAAAGGAGCCACCTGCCTTCTTCCCTATCCCATTGATCTCAAGCTACCTCGGGAGGTTTCCGAAACAAGGACCCCGTGGTTTGTAGGGCGCCCCCACTGGTTCGTCTAGGGTTGAGCTGGCCCAGCTCCTGCAGGGTCCCTGAAGTGGGCTTCCCAGGCCCAGGCGGTTGGGAGAGGCCTAGCAGACAGCTTCGGGTCCAGAGCATCCTCCAGCCGCTCCGCAACAGGAGCAAAACAAGAGCACGCGCACCTGTCGGCGCCCCGCCCCCTCCGCCCGGCCCAATCGTGGGCAGGGGCGGGGCCGGGGCGC**AGTCGGAACCCGCGCAGAGCGCCGCGGAGGAGCAGGAGCAAGCGCAGCCTCGTTCCTGCAACCTCGTCCCCGCCGCCCGCAGACTTGTCTCCACTGCCGCTGCCGCCGCCGCCCCCGGGGC**

Table S1. Prediction of transcription factor binding sites in Abcg1 promoter region.

| **Matrix ID** | **Name** | **Score** | **Relative score** | **Sequence ID** | **Start** | **End** | **Strand** | **Predict sequence** |
| --- | --- | --- | --- | --- | --- | --- | --- | --- |
| MA0144.1 | Stat3 | 11.0304 | 0.8908 | Abcg1 promoter | -341 | -332 | + | TACCTGGAAG |
| MA0144.1 | Stat3 | 7.2431 | 0.8212 | Abcg1 promoter | -710 | -701 | + | TTCTAGGAGA |
| MA0144.1 | Stat3 | 6.7592 | 0.8123 | Abcg1 promoter | -773 | -764 | + | TGCTAGGGAG |

**>Mouse Abca1 Promoter (-3255-1) (mouse Abca1, Gene ID: 11303)**

TCAGGAAGGATTCCTGAGCAGCCCCATGGAGGCCCAAACTTGTGGAGGAACTAGGCAGGCTCACAGATCTGGAGAAAGGGTTG**TTCTGGGAAA**AGGAACAGGACAGTGTAGTCCAAGGCCTAAGAGAGAGTGAGTGCAGCTTGTCCCTAGGGGGAGTCCTCCAGGGCATGGCCAAGGAGGTGAAAGGAGGTGTTCCTTGCTGAAGGCCCACCAGGGAAGTTGAGGAAACCTTGGGGAGGACTCCATCCAGGG**TGGCAGGAAG**CTGTTGAAGGGTAGACAAGTACCATGTGTAGATATTTGATTTTAGTCAAATAGGGGTGCTCTTGTGTCCAAGGGAGGAGAATGGGGCAAGCCTGGAGCCAAGGAGGCCAATTTGGAGTTGCTACTCTAGTCACGTAGGATCAAGAGAACGGTTCTTGATGGGTGAAGTTTCTAAAGTGGGCTCGCTTCCAGCCAG**TGTCTGGAAA**TTCATGCAGGGGCAGGAGCAAGCGTGTTATACTTCAGAACATTGAGAAATCCAGCGCACAGAACTGCCTTTCCCCTTGTGCTGTTGGAGGTTTGTTTGTTCACTTCCTTGGGAAACTGATGTGGAAATGTCCCATTGGCTTTTGTTACCTGGGTAGAAGGTATTTCCAATCACTTTGGGAGGAACATTGCCCTCAGCATGGATCTCTATGTTCAGAGATGGGGGTATGGGTGAGAATTATGCAGGTGTCCAGGAGGAGGGGTGGGCTGCAACGTCACCGTCACTTGTTTCCGTTTTCACACCCAGAGAAGAACTTTTCCTCTAGTCTCATGACATTTGTGCTGTGTCCCTGGGGAGCCTGGAATCTGAAACTAGTTGCAGAGACTCAGAAACTCATCAAC**TTCTAGGAAA**ATGTCCTCCAAGAAAATCCAGTTTTTCGTCTCATCCCAAGGGCGCTCTGCAGGGCTGCGCTCTCTTCTCTCATGAGCACGGTGTGGGGCTCTGTCTCTATTTGCCCCTGAACATCTCTGCTCAGGAGTCAGGAAACAATTCCAGATATGGATTTGGCCCCAGAGAGTGACCCTAAGGTATTCAGAGCTTGTCCTTCCTACTTCATTCACCCCACCCCTCACTTTCAGAAACAAGAATGTCCTCTTTAGATAAGCAGCCGCGCAGCTGAGGGTGCGGGTGGGACCTGGGGACCTGGGTTTCTTTGTTATTTGCATTATGCCTAGCACTGCATTCTGAATTTATTTCTATCACCTCATTTAATACTATAAAATACCAGACAAGGGAGATACTACTGCCCCCTGTCGGAATGAGGAACTTGCTGCTCAGAGCCGATAAGATAAATCTTGTCCTAGGATCACAGTTGGGCATCTGGGAGCTAGTGCCTTCCAGGCTTCATTCTGAGGCTTTGTTTCCCTTTTGTTGCAGCACATTGATTGTATTTCAGCCTTTCTTCTATTTTACGGTGGGCTCGGAGAAAATTTCTGTACCCTATAAGTCAGCTAAGTGATTTCTGGAGAGTGGCTTTAACTGAAGAAAAACTCGGTTAATAATTCTTTAATATTTTAAGTGCCACTTAAAAACAATTTTTTCACCTCCCTGTCAGTGTTCTGAGTCCAGTTGGCATCTCTCAACAGGGAACCGCTAGCCAAATGGTACTGACTAGGTCTTCCTCTTGCTATTGGCCTAAGTCATCTTATTTTTGCAGTAAGGAGCTGTGGGTAGCCTGTGCCCTTTCTGGATTTGCTGAGCAACAATAAAGAAGACTCTAGGGTTTGACTACCATAGGATTTCCGGTTTATTACATCACTAATAAATGAGCTAGAATTAGCCGCTCTCGGGCACCCATTGTTCTGGACATGTTGATTTTTGTTTTCTTTTTCCTTCTTTCCTAGACATTTTTAATCTTAGTTCTCTTGTATCAGGGAACAAAAGGGTATCACAGGGACTTAAAATCCTATCAAAAACCTCCCACGCGTTGTTCAAGATCATGGGCTTGATGGGTGAAGTTTTTGGTTTTGTCTCTTAGCATTTCACCCTGATATTTCACCTTTGCACAACAGAAGGCTACTAGACGCCACAGTCACTGGGAGTCATTTCATAGCTTCATAGGTTTCTGCTGAGGGTTTGTGACTAGCAACCATTGCATGGTCCTGACTTTAATAATCCACGTGTCCCTCCTCCTCCTGGCACTTTTCAATTTCCCCTTAATCCTTAGCTTGCTTTAGCTGAAGTCTTTGGAAGTTTCTCAGCATTGAATAAATACAGCTGGGTAGTGGGTAATAAGGCCACTGGGTAAGGAAGACACAGGCTGTTGTACGACCTCCTTGTGAAAATCTCTCAGATGGTGAACGGTCCCCCCAGGGAGACCTTTGAGACATCTGTGTAGGAGTGAAGCGGCCATGTGTTCAGGTGTAGGTAGAGCAAGTCCAGAAGTGTGCTCCATATGTCCCAACTGTAGGAGCCTCCTGCGTTCACAGATGCCCTGCTGGGATCAGGCCTTGGCAGGCTCTCTCCTCTGTGGGATACTGTTTTCTTTCCACAGAGACACAACACAAGCTTTGTCCATATCACAAGAGAGCCAGGTGTCCGTGGCACTGCTCTGGTGCTTACGCATAGTCGTCTAAGACAGTCCTCAGCCTTCCACCTGGGGAAACTGCGTCGCAAATGATGCCGTTTATCACTTGAAGGGCTCAGCTTTGTCTGTAGCGAAATGTTAGAGAATATCACTAGTCACTCCAGGGATCAGGGAGAATTTATAAGTGCTGCTGATGGGTCTTGGGAGAAAACATATATTCATAGGGAAGGCAGGCTGGCCTCTCGGGCTCTTGGATGGCACGTCTGATCTTTGTTTTGGCCAGGGAAAGTTACTTTCTCATTCTTAAAATAATCTCAGTGGTGCTCATATGATGAGAACCATCGATTGCGTCTGACCAGCTGCCAGCCTATAGCCAGCAAGCCTGCCCCGGAAGCTCTGTCTGCTGGAGATTCTGGGGGTTGGCAGTCCAGGACTCATGACCTTTTGACAAGCCACCAGGGTGTCAGAGGTGTCTGGTGAGAGGGCTG

GAGCAGCAACTCTTCTCCGGCATAGGGCTTTGAAGCAGTGATTGACAGATCTCTCTCTCTCTCTCTCTCTCTCTCTCTCTCTCTCTCTCTCTCTCTCTCTCGTTTTATCTTTCAGTTAATGACCAGCCACAGAGTCACAGCTCTGTGCTCTGGCTGCTCCCTCCAGGGCTCTCGAGCCGCAGACGCAGGTCGCTGTGGGTGCCGGCTGTGGTGAC**ATGGCTTGTTGGCCTCAGTTAAGGCTGCTGCTGTGGAAGAATCTGACATTTCGAAGGAGACAAACA**

Table S2. Prediction of transcription factor binding sites in Abca1 promoter region.

| **Matrix ID** | **Name** | **Score** | **Relative score** | **Sequence ID** | **Start** | **End** | **Strand** | **Predict sequence** |
| --- | --- | --- | --- | --- | --- | --- | --- | --- |
| MA0144.1 | Stat3 | 13.0169 | 0.9272 | Abca1 promoter | -2388 | -2379 | + | TTCTAGGAAA |
| MA0144.1 | Stat3 | 11.9314 | 0.9073 | Abca1 promoter | -3172 | -3163 | + | TTCTGGGAAA |
| MA0144.1 | Stat3 | 9.9880 | 0.8716 | Abca1 promoter | -3003 | -2994 | + | TGGCAGGAAG |
| MA0144.1 | Stat3 | 8.0404 | 0.8359 | Abca1 promoter | -2798 | -2789 | + | TGTCTGGAAA |

**Table S3. Sequences of the primers used for quantitative PCR analysis.**

| Gene | Forward (5’ 🡪 3’) | Reverse (5’ 🡪 3’) |
| --- | --- | --- |
| *LXN* | GCGGTTATGTAATGTGGCAG | AATGTCGTGGAGTAGAATGGTG |
| *Abca1* | GGTTTGGAGATGGTTATACAA | CCCGGAAACGCAAGTCC |
| *Abcg1* | GGGAAGTTGATAAAGGATGT | GATTCGGGCTATGTATGG |
| *CD36* | TGGAGCTGTTATTGGTGCAG | TGGGTTTTGCACATCAAAGA |
| *SR-A* | AAAGAAGAACAAGCGCACGTGG | GAGCACCAGGTGGACCAGTTTG |
| *IL-6* | TACCACTTCACAAGTCGGAGGC | CTGCAAGTGCATCATCGTTGTTC |
| *iNOS* | GTTCTCAGCCCAACAATACAAGA | GTGGACGGGTCGATGTCAC |
| *Arg1* | CTCCAAGCCAAAGTCCTTAGAG | AGGAGCTGTCATTAGGGACATC |
| *TNF-α* | TGACAAGCCTGTAGCCCACG | TTGTCTTTGAGATCCATGCCG |
| *Gapdh* | AGGTCGGTGTGAACGGATTTG | TGTAGACCATGTAGTTGAGGTCA |
| *IL-1β* | TGCCACCTTTTGACAGTGATG | TGATGTGCTGCTGCGAGATT |
| *MCP-1* | TTAAAAACCTGGATCGGAACCAA | GCATTAGCTTCAGATTTACGGGT |
| *CD206* | GGGTTGCTATCACTCTCTATGC | TTTCTTGTCTGTTGCCGTAGTT |

**
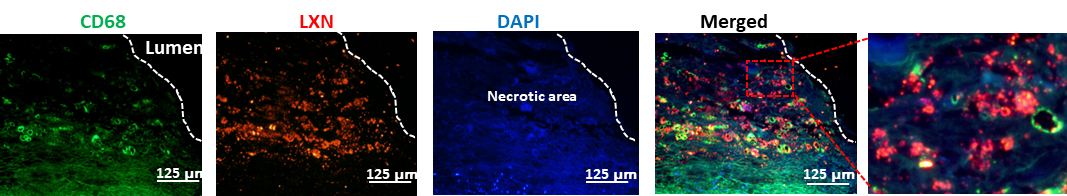
**

**Fig S1. Immunofluorescence staining of LXN and CD68 in the necrotic area of human atherosclerotic plaque.** (CD68, green; LXN, red; DAPI, blue). Scale bar, 125 μm.

**
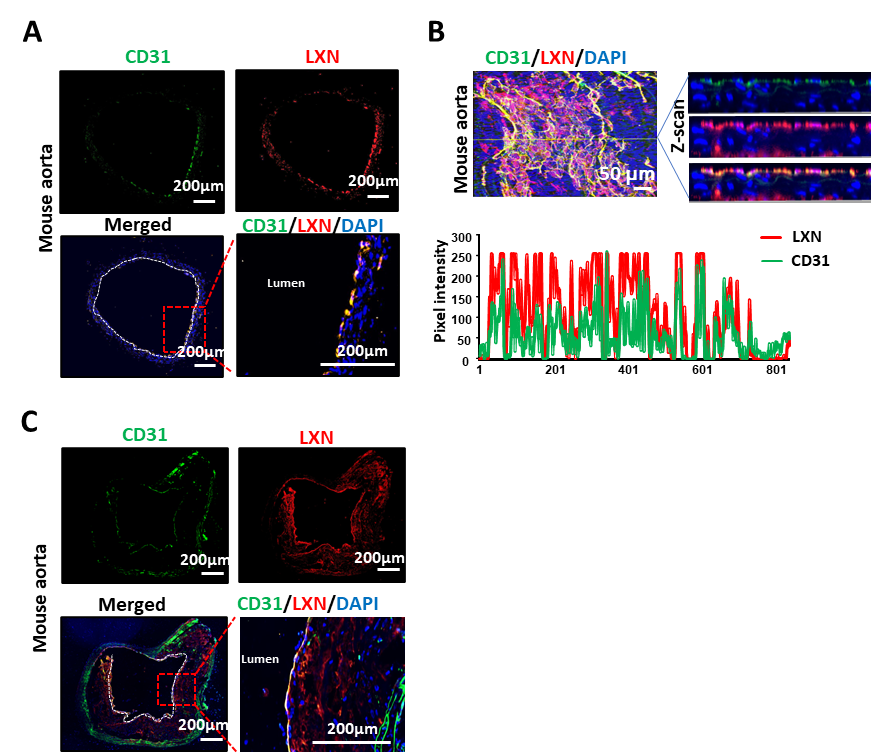
**

**Fig S2. The distribution of LXN in normal and atherosclerotic blood vessels of mice. (A)** Immunofluorescence staining of LXN (red) and CD31 (green) in aortas from AopE^-/-^ mice fed normally. **(B)** Representative Z-scan image of *en face* Oil Red O-stained aortas from AopE^-/-^ mice fed normally. **(C)** Immunofluorescence staining of LXN (red) and CD31 (green) in aortas from AopE^-/-^ mice fed HFD for 12 weeks.

**
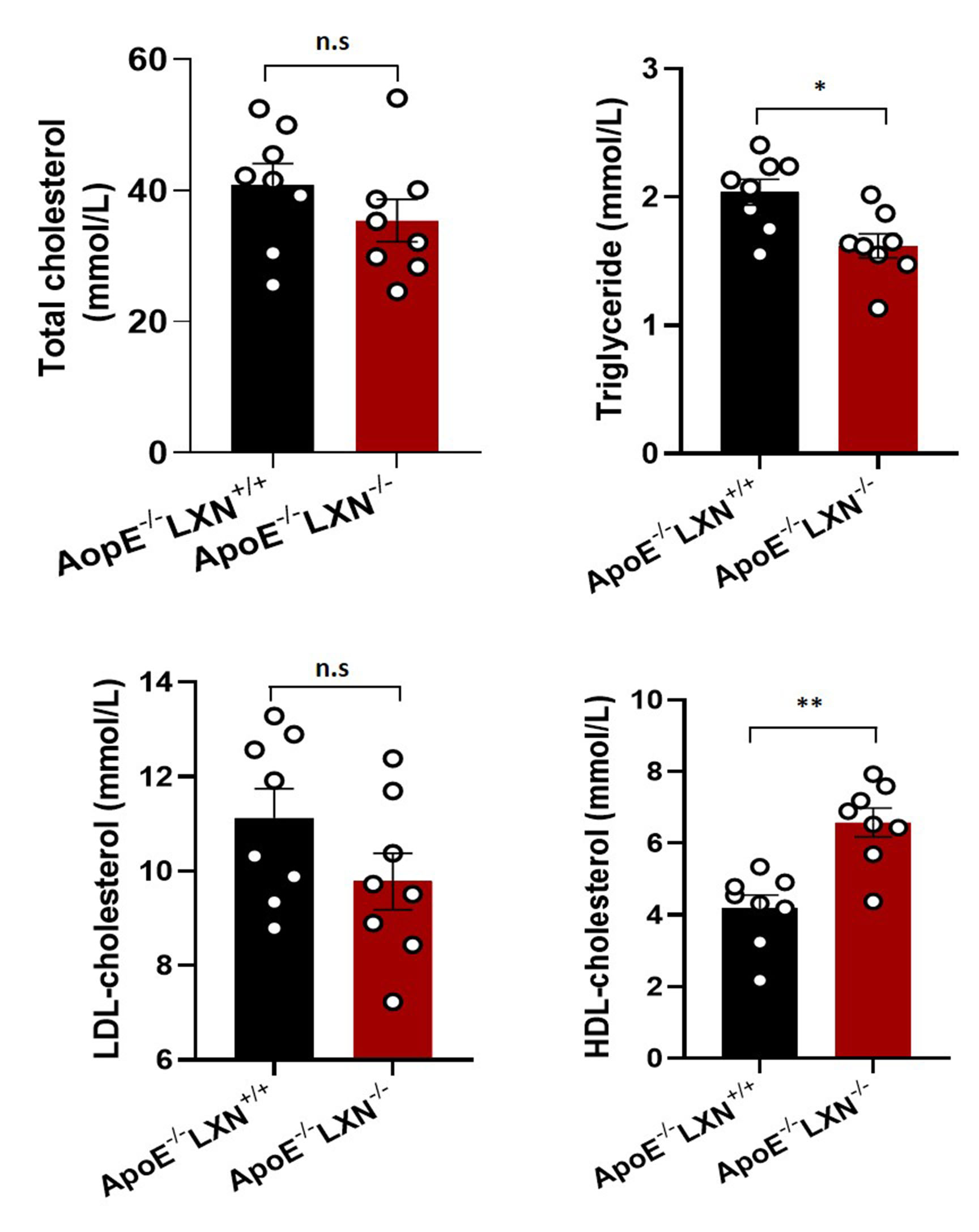
**

**Fig S3. Effect of *LXN* deletion in *ApoE^-/-^* mice on lipid metabolism.** *ApoE*^-/-^*LXN*^+/+^ and *ApoE*^-/-^*LXN^-/-^*mice were fed a HFD for 12 weeks (n=8). Serum TG, total cholesterol, LDL-C and HDL-C levels were determined by ELISA. Data are presented as mean ± SEM. The 2-tailed unpaired Student t-test was used for statistical analysis. **P* < 0.05, ***P* < 0.01.

**
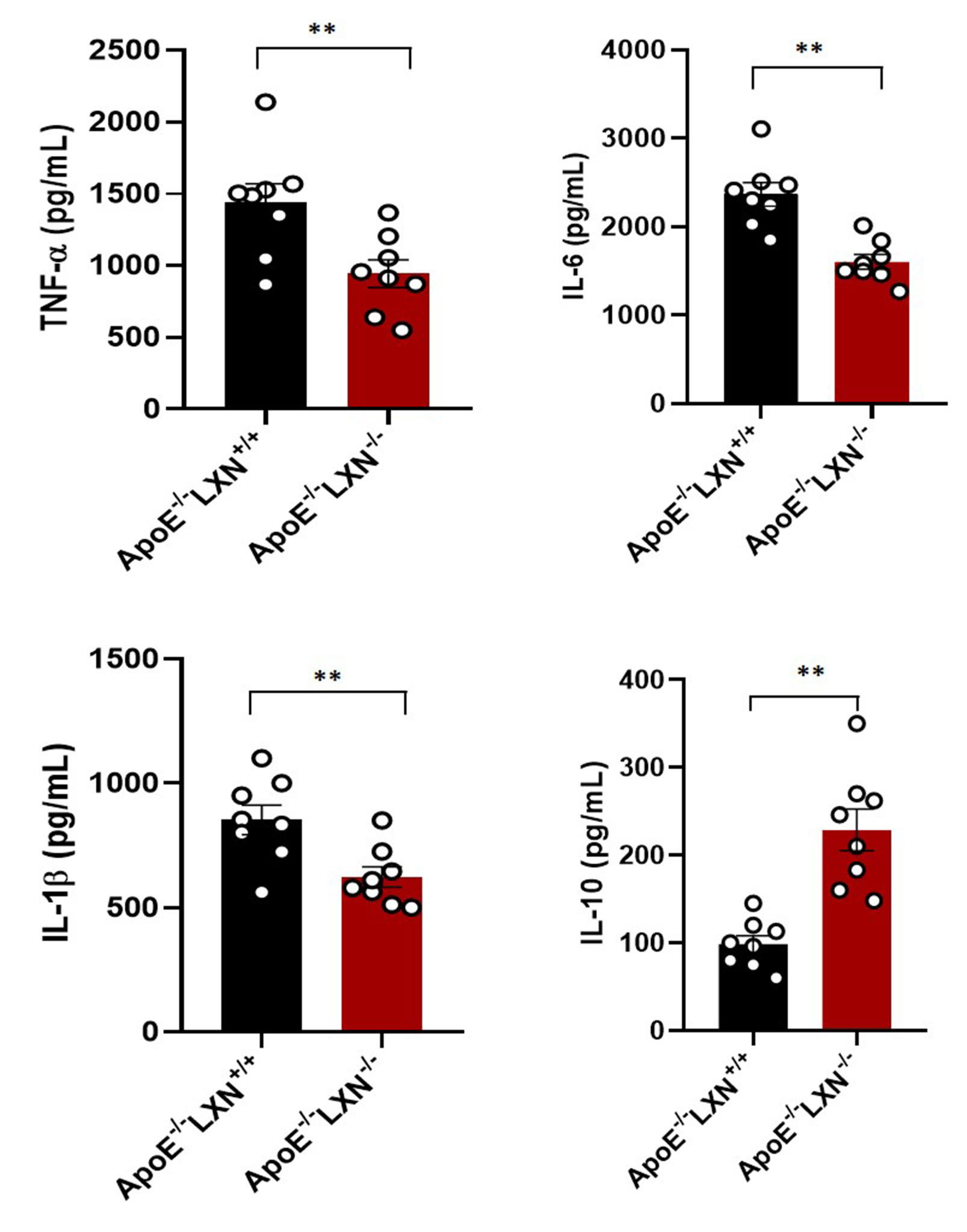
**

**Fig S4. Effect of *LXN* deletion in *ApoE^-/-^* mice on inflammation.** *ApoE*^-/-^*LXN*^+/+^ and *ApoE*^-/-^*LXN^-/-^*mice were fed a HFD for 12 weeks (n=8). Serum TNF-α, IL-6, IL-1β and IL-10 levels were determined by ELISA. Data are presented as mean ± SEM. The 2-tailed unpaired Student t-test was used for statistical analysis. ***P* < 0.01.

**
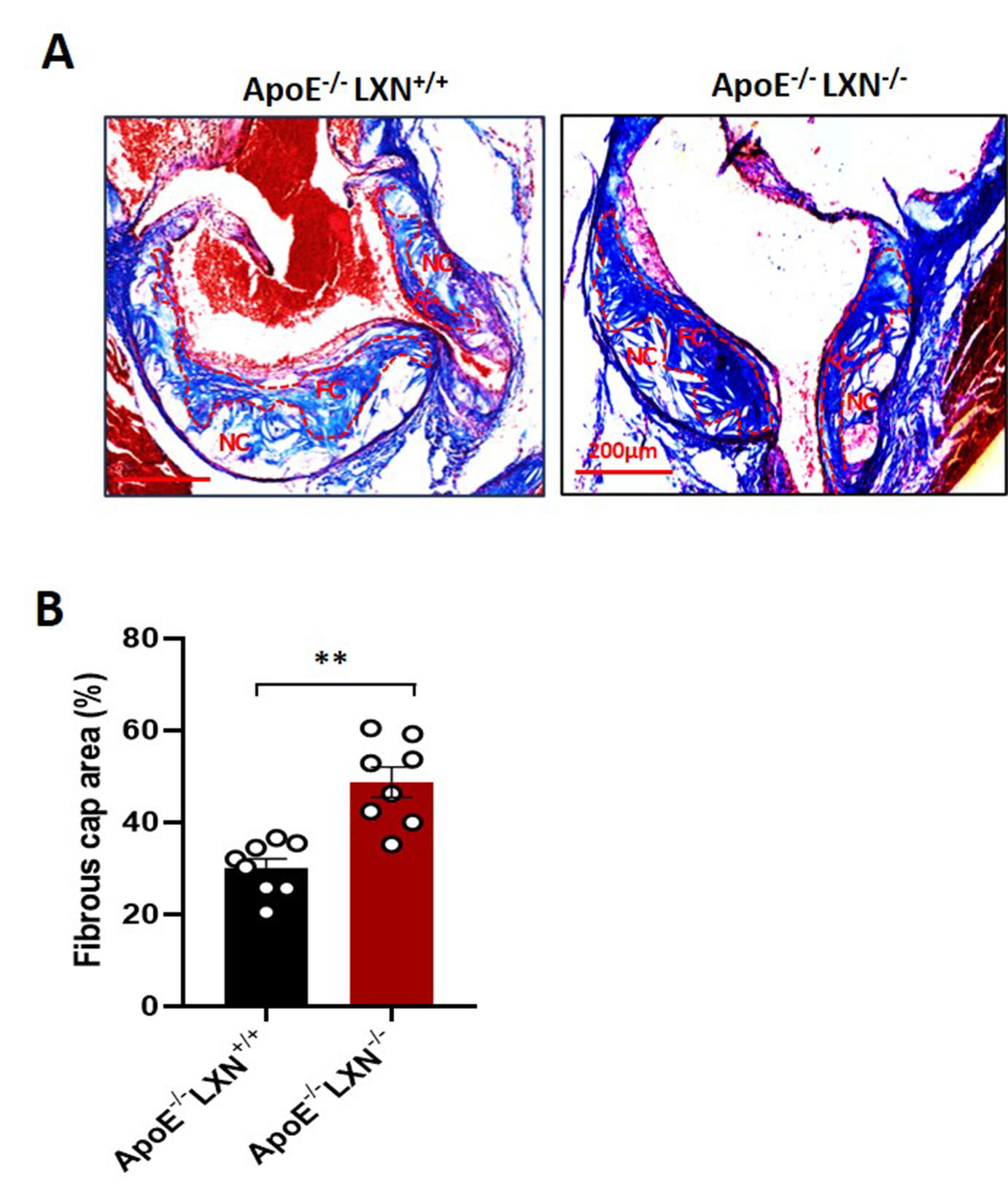
**

**Fig S5. Effect of *LXN* deletion in *ApoE^-/-^* mice on atherosclerotic plaque fiber cap.** *ApoE*^-/-^*LXN*^+/+^ and *ApoE*^-/-^*LXN^-/-^*mice were fed a HFD for 12 weeks (n=8). The sections of the aortic root were performed, and the collagen contents in the lesion was performed by staining with a Masson’s trichrome staining Kit. Mark the fibrous cap area (FC) and necrotic area (NC), and calculate the fibrous cap area ratio of the lesion area. Data are presented as mean ± SEM. The 2-tailed unpaired Student t-test was used for statistical analysis. ***P* < 0.01.


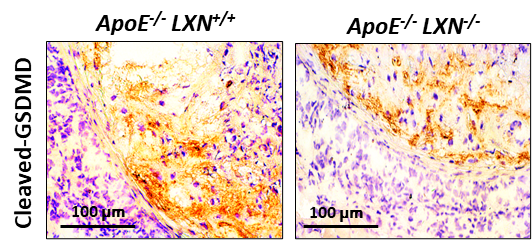


**Fig S6. Immunohistological analysis of Cleaved-GSDMD expression in atherosclerotic plaques in AopE knockout and LXN/ApoE-double knockout mice.**

**
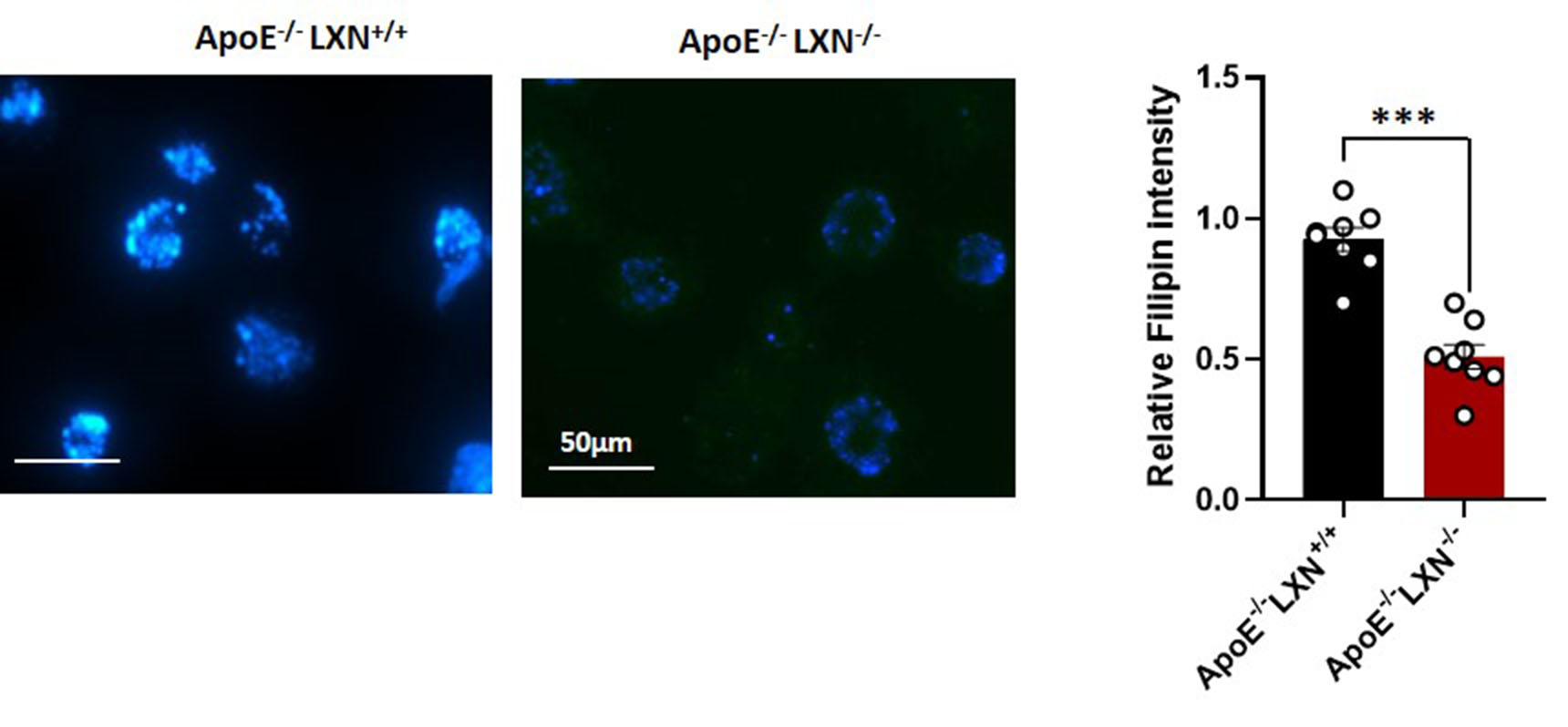
**

**Fig S7. Macrophage loss of *LXN* promotes cholesterol efflux.** *ApoE^-/-^LXN^+/+^* and *ApoE^-/-^LXN^-/-^*BMDMs cultured in six-well plate and treated with 20 μg/mL ox‑LDL for 48h. The cells were washed with PBS for 3 times and then stained with Filipin staining kit. Take fluorescent photos and calculate the relative Filipin intensity. n=8, Data are presented as mean ± SEM. The 2-tailed unpaired Student t-test was used for statistical analysis. ****P* < 0.001.

**
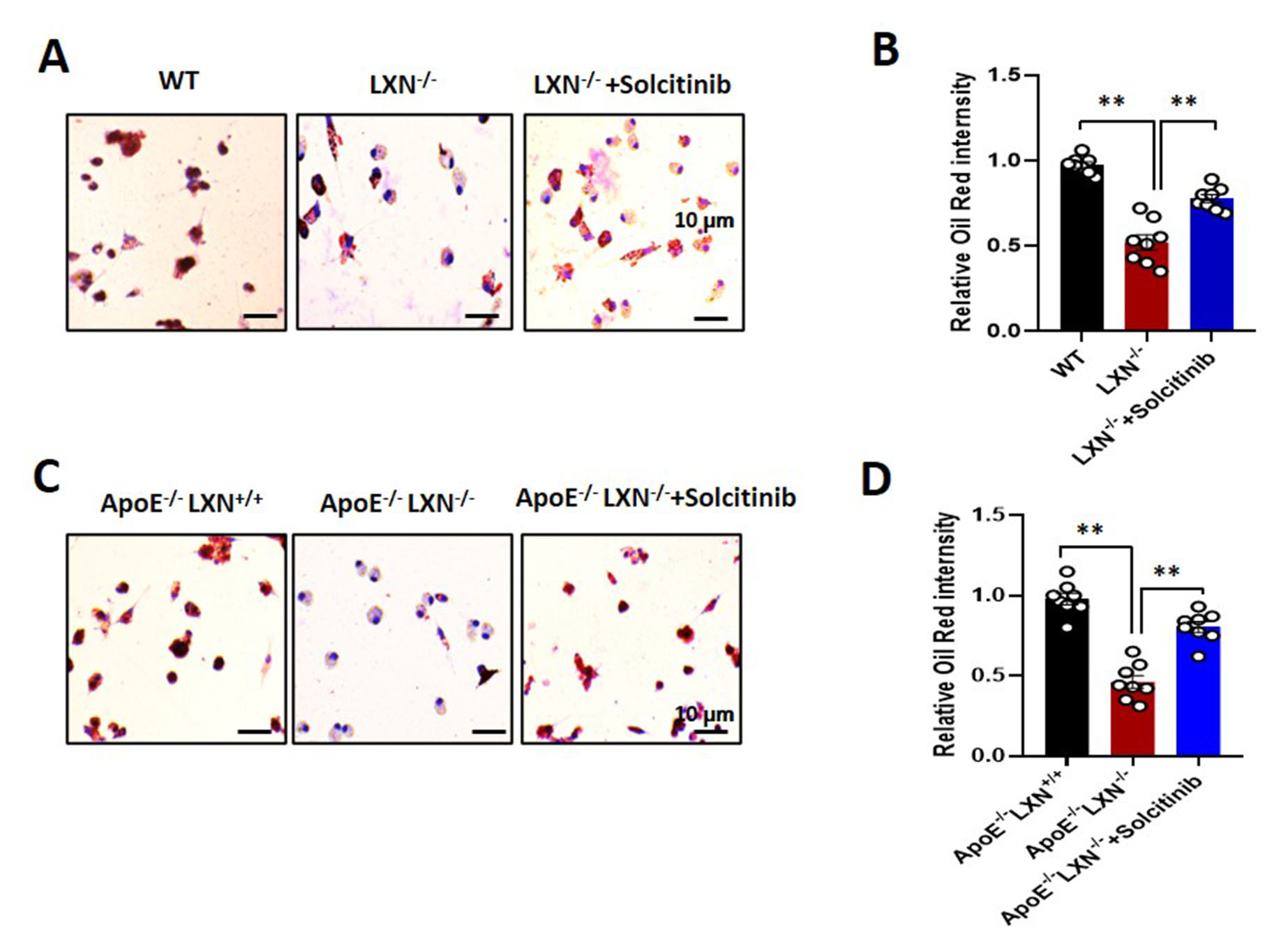
**

**Fig S8. JAK1 inhibitor can reverse the reduction of foam cell formation caused by *LXN* deletion in macrophage.** Peritoneal macrophages (PMs) are isolated from WT and *LXN^-/-^*mice (A, B), *ApoE^-/-^LXN^+/+^* and *ApoE^-/-^LXN^-/-^* mice (C, D), respectively. PMs are incubated with 20 μg/mL oxLDL in the presence or absence of 20 ng/mL solcitinib for 48h. Cells were fixed with 4% formaldehyde and stained with Oil Red O. n=8, Data are presented as mean ± SEM. One-way ANOVA was used for statistical analysis. ***P* < 0.01.

**Uncropped scans of membranes and gels used in the main figures**

**
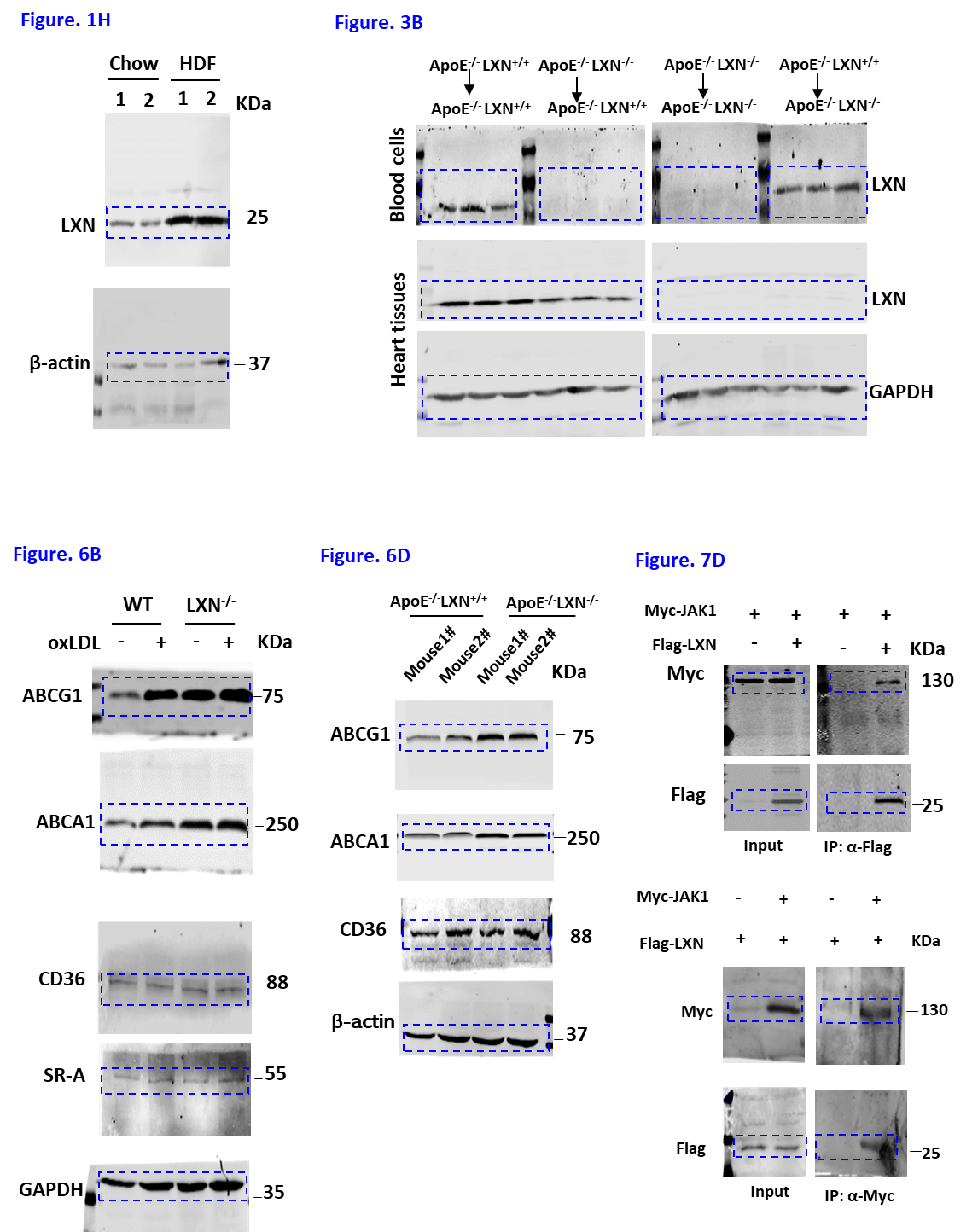
**


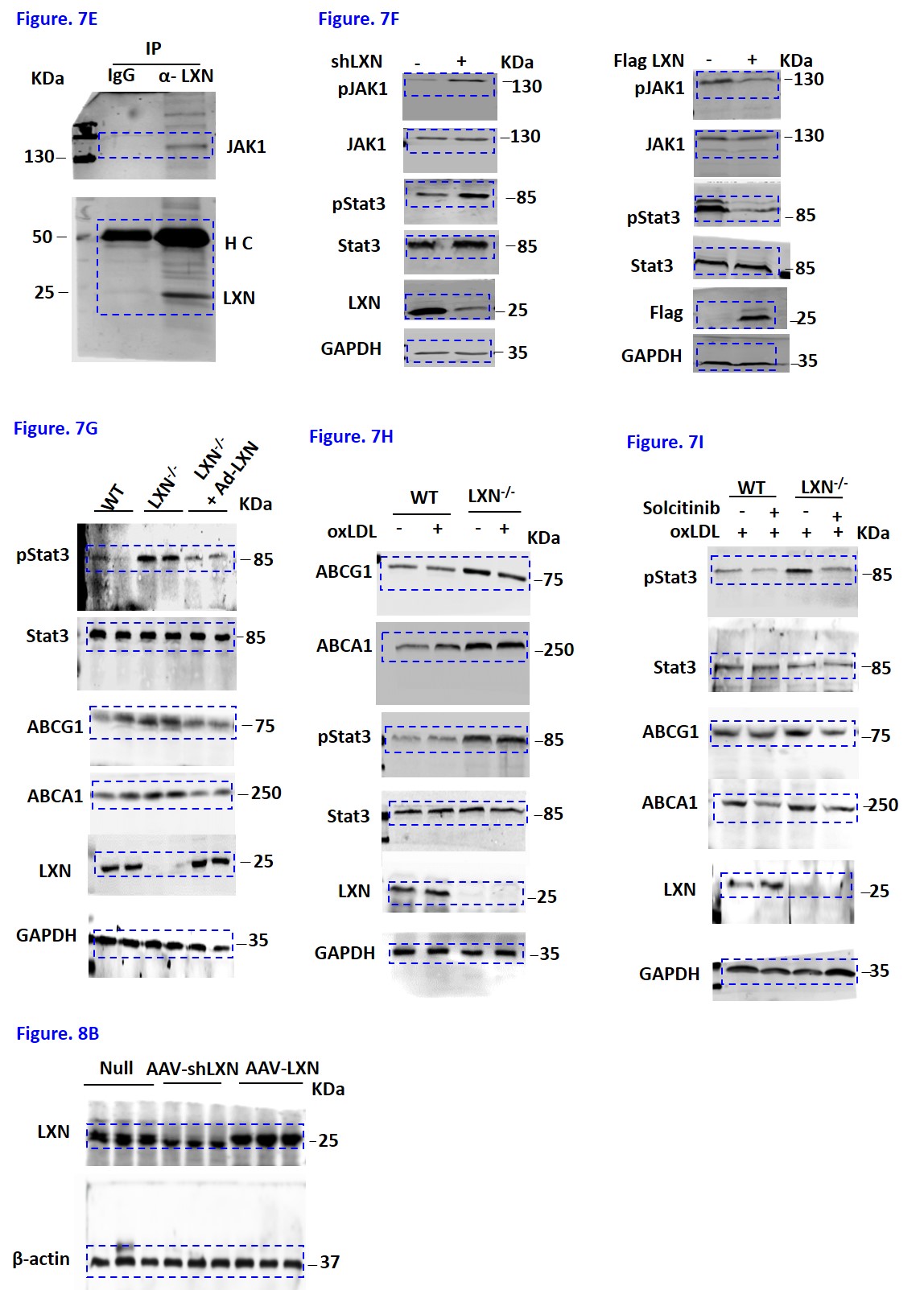

Supplement: Supplementary file 1 — Supplemental data [file 41419_2024_7141_MOESM1_ESM.doc]
